# Stories of a water-table: anomalous depressions, aquitard breaches and seasonal implications, Shelby County, Tennessee, USA

**DOI:** 10.1007/s10661-023-11531-z

**Published:** 2023-07-15

**Authors:** Daniela Lozano-Medina, Brian Waldron, Scott Schoefernacker, Anzhelika Antipova, Rodrigo Villalpando-Vizcaino

**Affiliations:** 1 Center for Applied Earth Science and Engineering Research, The University of Memphis, Memphis, TN 38152 USA; 2 Department of Earth Sciences, The University of Memphis, Memphis, TN 38152 USA

**Keywords:** Water-table surface, Water levels, Water level seasonal change, Cokriging, Aquitard, Inter-aquifer exchange

## Abstract

An extensive water level survey of the water-table aquifer (i.e., shallow aquifer) within Shelby County, Tennessee, was conducted in the dry (fall 2020) and wet (spring 2021) seasons. Water-table surfaces were generated using cokriging to observe seasonal differences to identify anomalous water-table depressions, indicative of an underlying aquitard breach. Seasonal differences were attributed to non-coincident control and timing between the surveys and when optimum dry (fall) and wet (spring) conditions existed, as observed through comparisons with continuous historical water levels from 12 shallow monitoring wells. Additionally, data from fall 2020 were compared to previous studies in 2005 and 2015 to determine decadal changes in levels and shape of the water-table surface which were mostly attributed to changes in data control and potential climate variations. A prediction error map was generated from the 2020 dataset to identify areas of the county with high-prediction error (>7.0 m) to offer guidance on where future well control would be optimal.

## Introduction

Groundwater is an important source of drinking water in many parts of the world, and understanding its flow and fluctuations within a hydrogeological system is crucial to protecting this critical resource. Water-level monitoring allows a glimpse of where the water is and where it is moving to. A less common benefit is found in stressed aquifers that are impacted through inter-aquifer water exchange which is common and naturally occurring. This leakage can be exacerbated when preferential flow paths exist through natural breaches in an aquitard, allowing for modern water to infiltrate into an underlying aquifer causing water quality concerns. Water levels can show the areas where this preferential exchange occurs beneath the surface (Bradshaw, 2011; Konduro-Narsimha, 2007; Ogletree, 2016). An example can be found in the multi-layered aquifer system of the Mississippi embayment in Shelby County, Tennessee, where the presence of aquitard breaches has been investigated for decades (Brahana & Broshears, 2001; Carmichael et al., 2018; Criner et al., 1964; Graham & Parks, 1986; Kingsbury & Parks, 1993; Konduro-Narsimha, 2007; Larsen et al., 2003, 2013; Ogletree, 2016; Parks, 1990; Schoefernacker, 2018; Torres-Uribe, 2020; Waldron et al., 2009).

Significant withdrawals for municipal and industrial uses have caused substantial water-level declines (>35 m) in the Memphis aquifer, the semi-confined aquifer that is bounded above by a confining unit that underlies the water-table aquifer and is the primary water source for this region since the late 1800s (Brahana & Broshears, 2001; Criner & Parks, 1976). This previously mentioned decline has resulted in a downward vertical gradient where water from the unstressed water-table aquifer finds preferential leakage paths through breaches in the intervening aquitard between these two aquifers (Brahana & Broshears, 2001; Criner et al., 1964; Criner & Parks, 1976; Graham, 1982; Kingsbury, 1996; Parks & Carmichael, 1990; Waldron & Larsen, 2015). Given that the water-table aquifer is more susceptible to contamination from anthropogenic sources due to its unconfined condition and is of lesser water quality than the Memphis aquifer, identifying aquitard breaches between these two aquifers is paramount.

A valuable product of collecting water levels in the water-table aquifer (or shallow aquifer) is the development of a water surface where anomalous depressions can help identify these hidden breaches since pumping from the water-table aquifer is limited. Another valuable use is their incorporation into ongoing numerical modeling of the area’s groundwater resources (Clark & Hart, 2009; Torres-Uribe, 2020; Villalpando-Vizcaino et al., 2021). Hence, this investigation seeks to (1) map water levels in the water-table aquifer; (2) identify potential aquitard breaches; (3) address seasonal water-level fluctuations; and (4) provide data for the calibration of the Shelby County numerical groundwater model. In addition, this research aims to illustrate the importance of data control by identifying high error areas within the study area, and appropriate data acquisition timing through the analysis of long-term water-level data recorded by pressure transducers.

### Study area

**Fig. 1:**
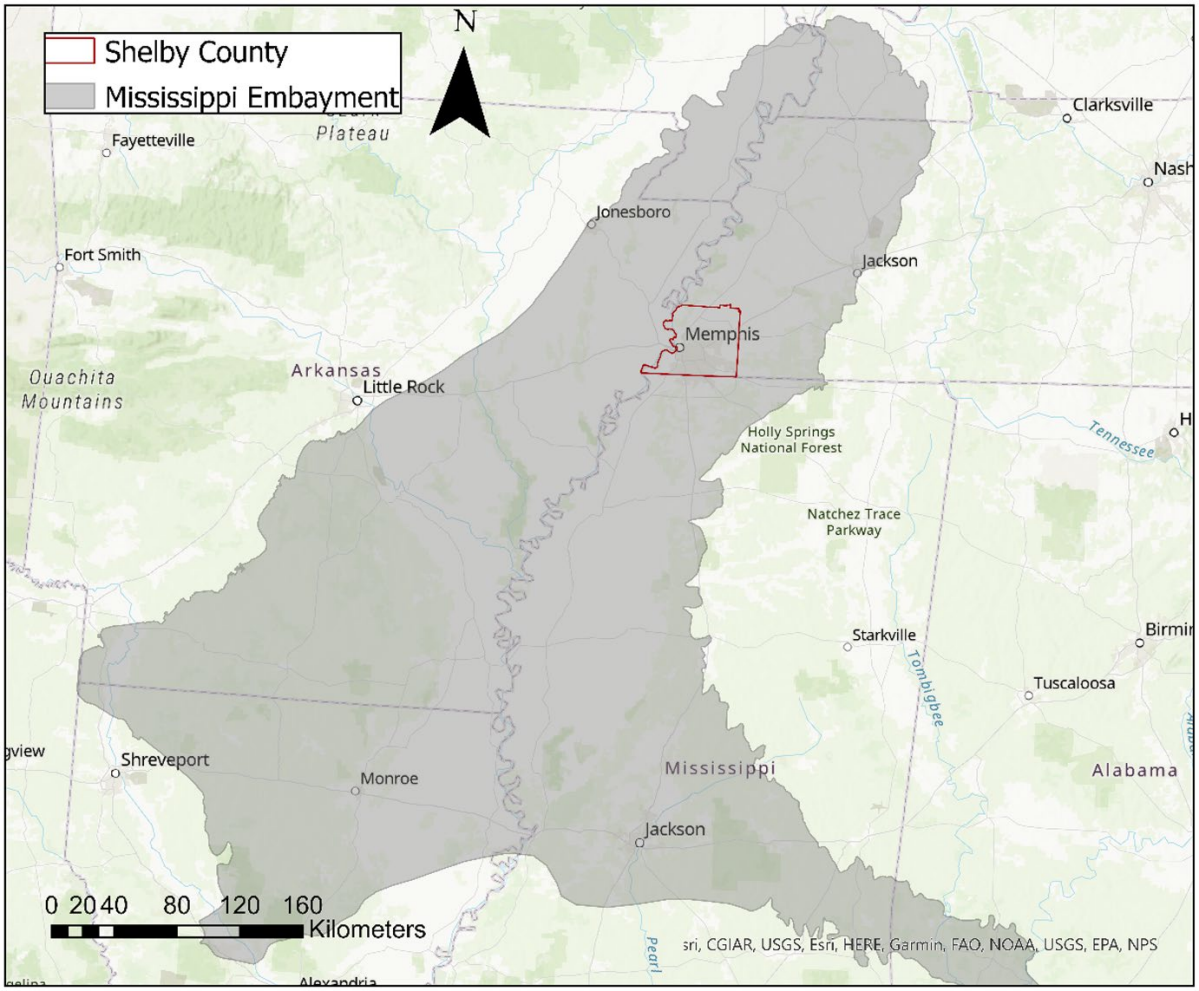
Location of Shelby County within the Mississippi embayment aquifer system (Clark & Hart, 2009)

**Fig. 2:**
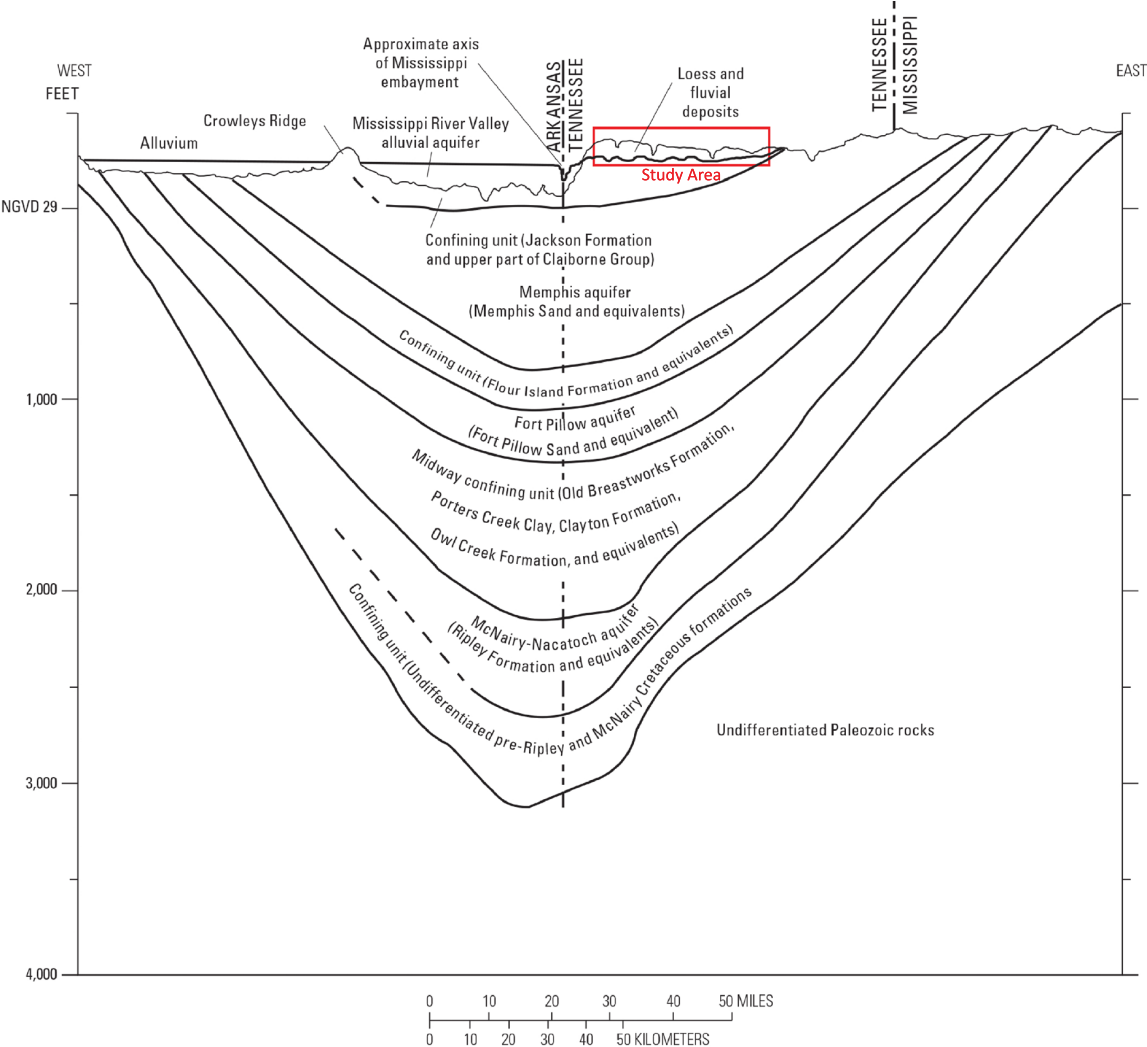
Hydrostratigraphic units of the Mississippi embayment underlying Memphis, Tennessee (Carmichael et al., 2018). Additional hydrostratigraphic units are shown below the Fort Pillow aquifer but are not discussed in this study

The Mississippi embayment is a collection of unconsolidated aquifers and aquitards that underlies portions of eight states in the south-central United States (Clark & Hart, 2009; Graham & Parks, 1986; Waldron et al., 2011) (Figs. 1 and 2). Located within the embayment is Shelby County, Tennessee, which solely relies on groundwater for public supply, with a total withdrawal of 696,000 m^3^/day in 2015 (Dieter et al., 2018).

**Fig. 3:**
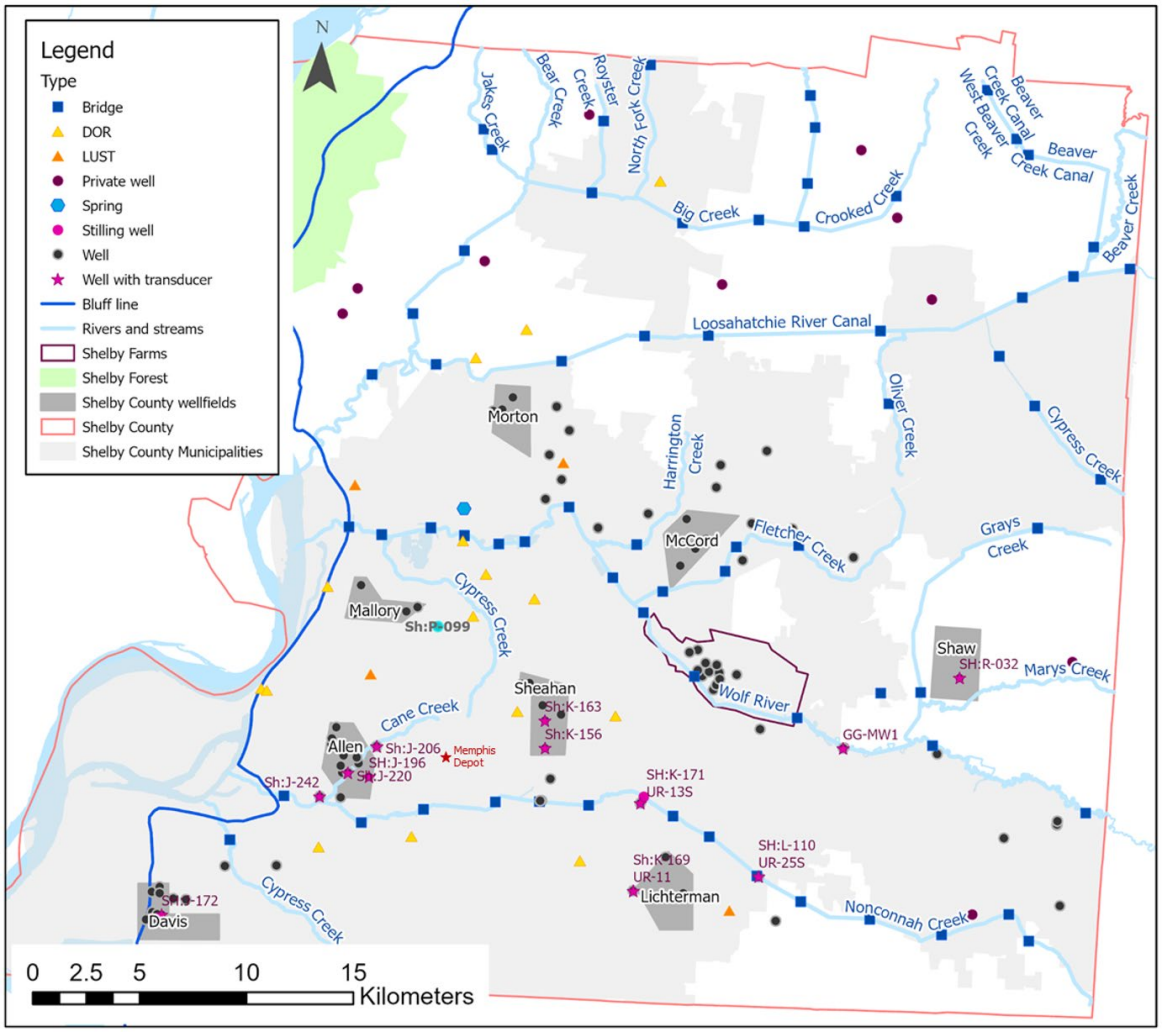
Water-level network locations with public and private wells, surface water features, TDEC sites, and flowing springs

There are three primary freshwater aquifers in Shelby County: the water table, Memphis, and Fort Pillow aquifers (Fig. 2). The water-table aquifer ranges in thickness from 0 to 30 m and is comprised of alluvial and fluvial deposits throughout the county (Brahana & Broshears, 2001; Graham & Parks, 1986; Konduro-Narsimha, 2007; Parks & Carmichael, 1990). The water-table aquifer includes the Mississippi River valley alluvial (MRVA) aquifer located westside of the bluff line (see Fig. 3) (Lloyd and Lyke, 1995); however, the MRVA aquifer is not investigated in this study since the research is conducted on the east side of the bluff line. The water-table aquifer in the eastern portion of the county corresponds to the unconfined area of the Memphis aquifer (Parks, 1990; Urbano et al., 2006). The water-table aquifer supplies water to some domestic and farm wells (Parks, 1990; Waldron et al., 2011) although high-capacity pumping in the water-table aquifer is limited or non-existent.

The water-table aquifer is underlain by the Upper Claiborne confining unit (UCCU), ranging in thickness from 1 to 61 m (Larsen et al., 2016). The UCCU is comprised of the Cockfield and Cook Mountain formations (Larsen et al., 2003, 2016) and acts as an aquitard, limiting the downward vertical water exchange between the water-table and Memphis aquifers (Graham & Parks, 1986), except for those areas, termed “breaches,” where the UCCU is either absent or thinning, or where fault-related connections exist.

Underlying the UCCU is the Memphis aquifer which is composed primarily of sand, with some clay and lignite, and ranges from 122 to 274 m thick (Larsen et al., 2016). It is the most productive aquifer in the Memphis area providing approximately 95% of the groundwater used for domestic, industrial, and agricultural uses (Graham & Parks, 1986; Kingsbury, 1996). The underlying Flour Island confining unit separates the Memphis and Fort Pillow aquifers, which is another important aquifer to the area. Only the water-table aquifer and the Memphis aquifer, by proxy of suspected breach locations in the UCCU, are considered in this investigation.

## Methodology

The development of a water-table map requires the identification of measurement locations, various measurement procedures, data processing, and interpolation of the final water levels. Three prior investigations were performed in 1987 (Parks 1990), 2005 (Konduro-Narsimha, 2007), and 2015 (Ogletree, 2016) which are compared with the fall 2020 survey of this investigation. However, this effort builds upon prior measured locations from these studies and follows more closely the post-processing procedures developed by Ogletree (2016), which generated water-table maps using empirical Bayesian kriging incorporating ground elevation as a secondary variable.

All prior investigations took water-level measurements during the dry season (September to early November) at available wells screened within the water-table aquifer. Surface water measurements along major rivers and tributaries were collected assuming aquifer connection and mostly gaining conditions as suggested by Parks (1990). Konduro-Narsimha (2007) and Ogletree (2016) took physical measurements of stream surface elevations while Parks (1990) relied on historical U.S. Geological Survey (USGS) 7.5-min quadrangle elevation contours at stream crossings and any recorded water levels from a 40-year span, though Parks (1990) concluded that any physical changes over this 40-year span were insignificant.

### Data collection

This investigation collected water-level measurements at water-table monitoring and private wells in addition to surface water levels at bridge crossings following Konduro-Narsimha (2007) and Ogletree (2016). A compilation of historical water levels between 2016 and 2020, from wells screened within the water-table aquifer at monitored sites (e.g., Divisions of Underground Storage Tanks or Remediation, termed LUST and DOR, respectively), were obtained from the Tennessee Department of Environment and Conservation (TDEC) for both dry and wet seasons where available. Similar to Konduro-Narsimha (2007) and Ogletree (2016), these historical water levels were compiled and averaged to be incorporated to the dataset.

Unlike prior investigations, this study also performed a water-level survey during the wet season. The first water-level survey was conducted from mid-September to early October 2020. The second survey was conducted from late March to early April 2021. Following the USGS Groundwater Technical Procedures, depth to water was measured using the Solinst electric water-level meters (e-tapes) calibrated through the USGS Hydrologic Instrumentation Facility (HIF) program prior to the surveys (Cunningham & Schalk, 2011). Water levels were obtained from 99 wells throughout the county, usually located proximal to utility wellfields (Fig. 3), with some exceptions of isolated wells scattered throughout the county. Given the scarcity of public monitoring wells in rural areas of unincorporated Shelby County (see Fig.3), an assessment of privately owned wells was conducted. Approximately 60 private wells were identified from the Shelby County Health Department records as screened within the water-table aquifer, yet only nine were used for water-level measurements due to property access and well construction restrictions.

Direct connection between surface water bodies (i.e., rivers and tributaries) and the water-table aquifer was assumed to exist based on Parks (1990) and Larsen et al. (2013); therefore, water levels were collected from three main rivers in the area: the Loosahatchie River, Wolf River, and Nonconnah Creek, as well as their tributaries. Following the methodologies described by Konduro-Narsimha (2007) and Ogletree (2016), water-level measurements were obtained at stream-bridge crossings using previously defined benchmarks (i.e., pre-installed bridge railing plates) as the point of measure. In some cases, there were no pre-installed plates so a different point-of-measure was used.

Plate placement, which occurred during Konduro-Narsimha (2007), attempted to find minimal surface water displacement since bridge crossings can constrict flow and often have erosion control structures. The same was attempted when finding alternative measuring points. E-tapes were extended from the designated measuring points down to the water surface, watching for wind effects to ensure a vertical dropdown to the water surface. Though not ultimately used, water levels were also obtained from flowing springs in isolated parts of the county. Most of the springs, except for one, were in the Shelby Forest area (see Fig. 3). These measurements were later discarded from the final dataset as they are located within the MRVA aquifer west of the bluff line (Fig. 3), the western boundary for this study.

To minimize spatial and measurement inaccuracies, all accessed features (e.g., wells and river benchmarks) were surveyed using a survey-grade R2 Trimble global positioning system (GPS) unit. Spatial precision (*x*, *y*) was less than 1 cm with a vertical precision (*z*) less than 5 cm. The GPS unit accuracy was regularly tested against a U.S. Army Corps of Engineers’ first-order, grade A survey marker prior to surveying. As mentioned, historical water levels from sites monitored by the TDEC were obtained for the dry and wet season periods for the 5-year period, 2015 to 2020. Available data were averaged to a single value per site. A total of 22 leaking underground storage tank (LUST) and 111 Division of Remediation (DOR) sites were reviewed, resulting in four LUST and 18 DOR sites that met the criteria and were added to the dataset (see Fig. 3).

### Data processing

Collected water level elevations (Appendix 1, Table 7) were interpolated using cokriging. Kriging is a widely used interpolation method that estimates missing values based on the weighted average of the available data (Isaaks & Srivastava, 1991; Snyder, 2008). This interpolation scheme modifies the weights of clustered data so that grouped points with similar information are assigned a lower weight (Snyder, 2008). Additionally, and contrary to other interpolation methods, kriging estimates the standard interpolation error which can indicate where additional control is needed to reduce interpolation uncertainty (Olea & Davis, 1999; Theodossiou & Latinopoulos, 2006).

There are several kriging variations with differing approaches, including ordinary kriging, universal kriging, Bayesian kriging, and cokriging (Oyana & Margai, 2015). Given the sparsity and clustering of measurement points, cokriging was selected as the interpolation method for this study, similar to others (Ahmadi & Sedghamiz, 2008; Boezio et al., 2006; Chung & Rogers, 2012; Hoeksema et al., 1989; Ogletree, 2016; Li and Zhao, 2011). Cokriging minimizes the variance of the estimation error by using more than one variable to compute missing values (Isaaks & Srivastava, 1991). The primary variable used was water elevation, and the secondary variable was ground surface elevation (See Appendix 1). The correlation between these variables was confirmed by obtaining a Pearson correlation coefficient of 0.70 between the two variables, close to the 0.73 coefficient obtained by Snyder (2008). Ground surface elevation data were obtained from a 1-m LiDAR digital elevation model (DEM) of Shelby County generated in 2020 (CAESER, 2020).

The Pearson correlation analysis used the 1-m LiDAR; however, to better relate to the smallest sampling spatial resolution, a sensitivity test was conducted. The sensitivity test assessed the advantage of resampling the highly detailed 1-meter LiDAR DEM surface to a larger resampled size using a bilinear resampling technique. Following Desbarats et al. (2002), increasing the cell size to 90 m did not notably change the correlation coefficient of 0.70. Therefore, this resampled surface was used in the cokriging processing which reduced the cokriging processing time significantly.

The result of cokriging produces an interpolated surface, or raster, with a grid size that followed the methodology described by Hengl (2006) using Eq. 1. The square-grid size (*P*) is a function of an empirical constant, the study area (*A*) and the number of sampling points (*N*).

1 $$P=0.0791\sqrt{\frac{A}{N}}$$

Subsequent to creating rasters, contours were produced by first applying a smoothing algorithm to the rasters by averaging each cell with its surrounding neighbors within a 1 km radius so as to capture four neighboring cells. Then, contours were generated on a 3-m interval given the typical 10 ft interval used for the study area (Criner & Parks, 1976; Kingsbury, 1996, 2018; Parks, 1990).

Water level data for fall and spring were not normally distributed; therefore, both datasets were log transformed to better approximate a normal distribution using skewness (0) and kurtosis (3.0) as the indicators of following a normal distribution. When mapping the data in a three-dimensional space and projecting the trend of data on the *x*- and *y*-axes, the water-table data had a quadratic trend, and the ground surface had a linear trend. Knowing this, it was possible to remove the trend during the cokriging process (Table 1).

**Table 1:** Skewness and kurtosis of raw and log transformed data

	Raw data			Log transformed data		
	Fall	Spring	Topography	Fall	Spring	Topography
Skewness	0.6137	0.6346	0.2985	0.2712	0.3138	−0.0258
Kurtosis	3.3778	3.2882	2.6189	3.0337	3.0733	2.5358

Through investigation of the semivariogram, many models were displayed against the data to determine which best followed the trend of the data bins. Given the s-shape of the semivariogram, the Gaussian model was the most appropriate choice. Choosing to optimize the model fit via autocorrelation (i.e., automated within ArcGIS Pro®), the resulting cokriging parameters are provided in Table 2.

**Table 2:** Groundwater and ground elevation interpolation parameters

	Fall 2020		Spring 2021	
	Groundwater elevation	Ground elevation	Groundwater elevation	Ground elevation
Nugget	0.0003	0.001	0.0013	0.0019
Major range (m)	3981.72		5561.43	
Sill	0.008	0.01	0.007	0.0023
Lag size (m)	497.71		695.18	
Number of lags	12		12	
Maximum neighbors	15	10	15	10
Minimum neighbors	8	5	8	5
Sector type	1 sector		1 sector	
Average standard error (m)	4.35		4.34	

## Results and discussion

**Fig. 4:**
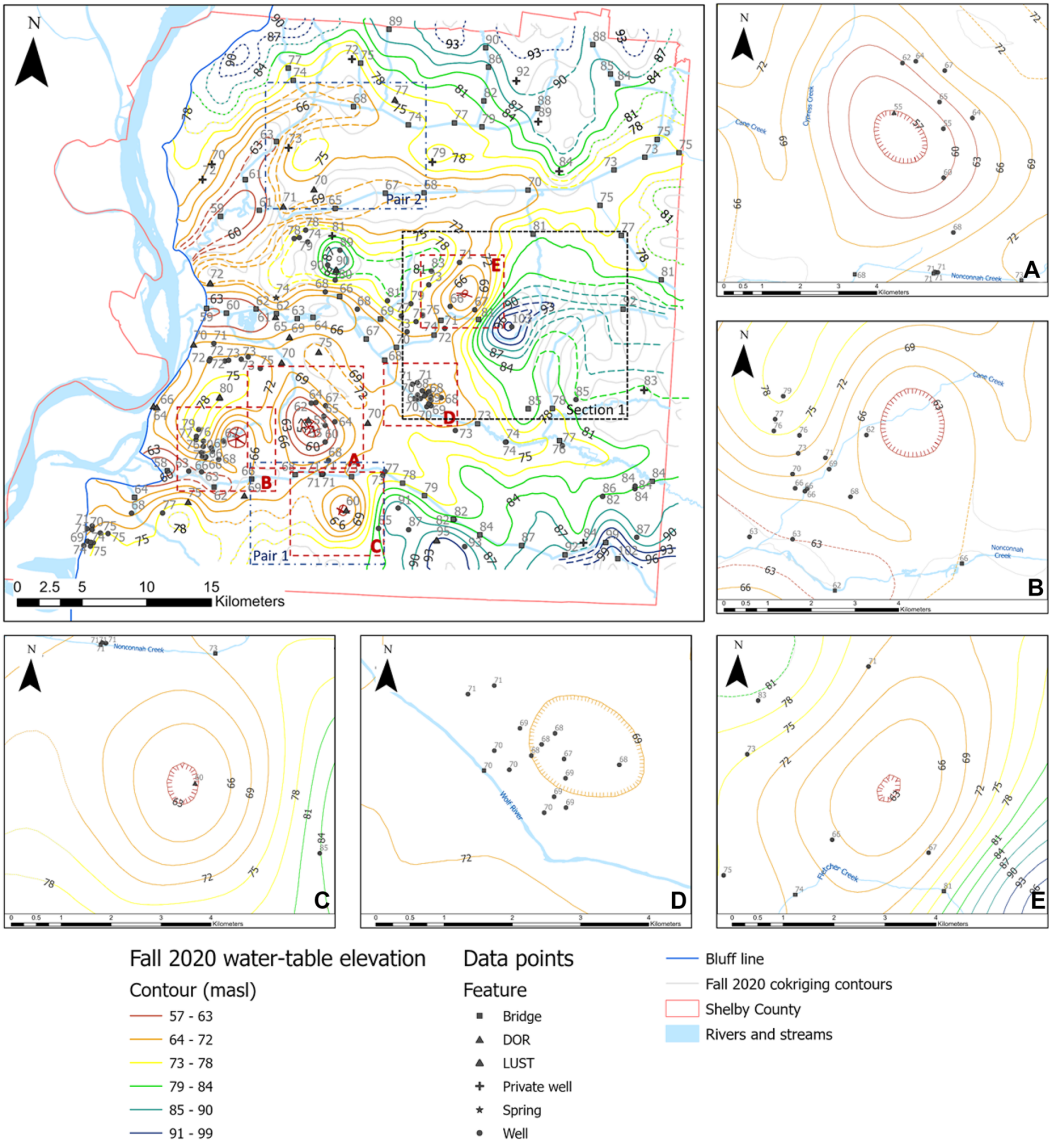
Fall 2020 water-table map. Gray lines represent original contours from co-kriging where dashed-colored contours represent manual adjustments proximal to streams. Dotted lines represent approximate locations. Dashed lines were manually modified. Hatched lines represent depressions. Dashed red boxes (A–E) represent water-table depressions shown in insets (A–E); pairs 1 and 2 blue dashed boxes are compared to Fig. 5 (spring 2021 water-table map); section 1 (black dotted line) compares to water-table maps from 2005 to 2015

**Fig. 5:**
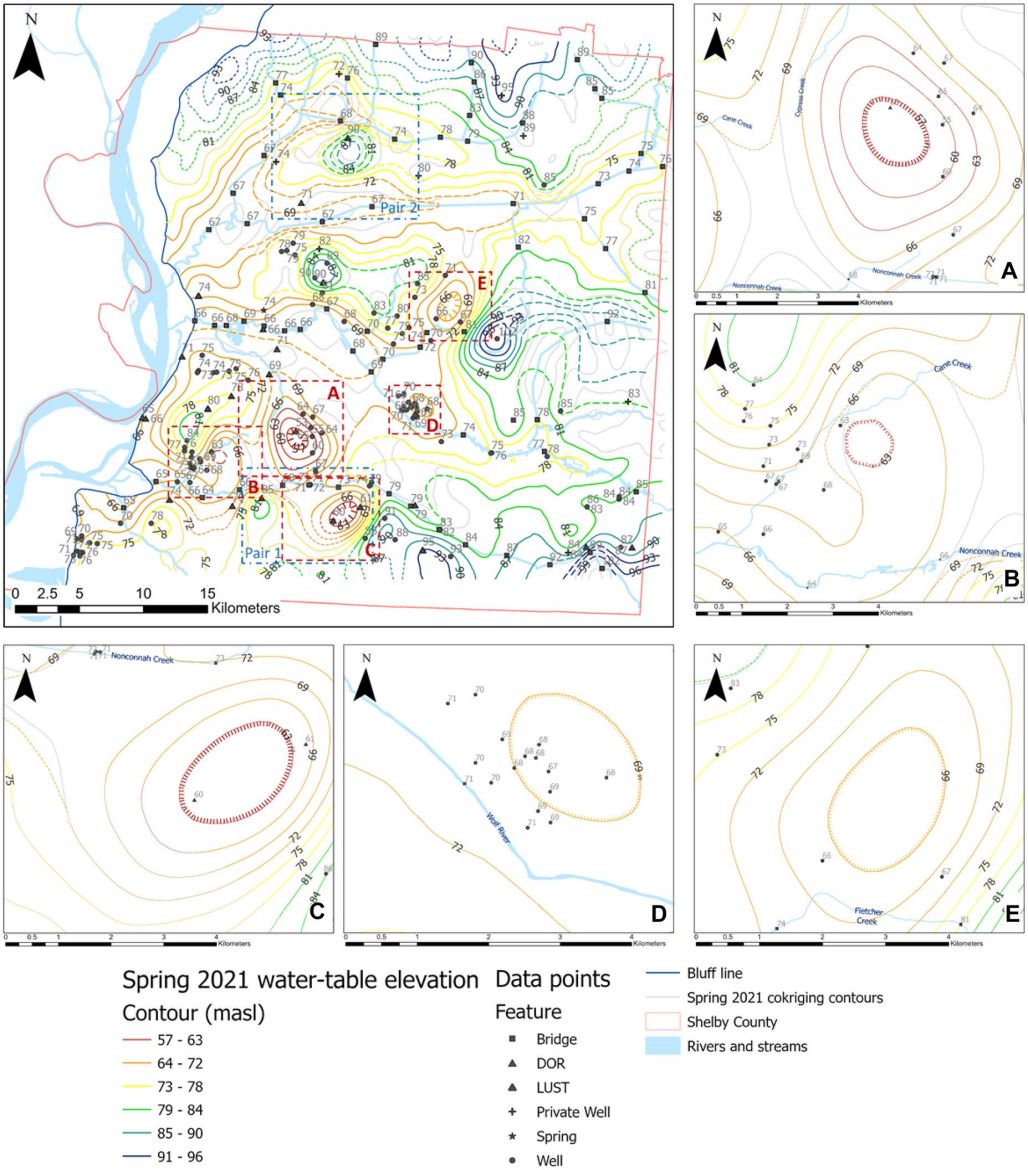
Spring 2021 water-table map. Gray lines represent original contours from co-kriging where dashed-colored contours represent manual adjustments proximal to streams. Dotted lines represent approximate locations. Dashed lines were manually modified. Hatched lines represent depressions. Dashed red boxes (A–E) represent water-table depressions; pair 1-2 in blue dashed boxes are compared to Fig. 4 (fall 2020 water-table map). (B) Insets of water-table depressions, same as boxes A-E.

The resulting grid spacing for fall and spring surfaces obtained from Eq. 1 was 210 m. Since cokriging does not identify and respect hydrologic boundaries, some contour lines were inaccurately crossing streams or forming depressions; therefore, contours were modified to follow a path that matched the assumption that the water-table is hydraulically connected to surface water. Although the existence of a breach under a stream is possible (Brunner et al., 2011; Sophocleous, 2002; Urbano et al., 2006), there are insufficient data to substantiate those artificial depressions. Figures 4 and 5 show the water-table maps for fall 2020 and spring 2021, respectively.

### Anomalous water-table depressions

An important outcome of developing a water-table map in this area is the indication of potential aquitard breaches reflected by anomalous depressions (Figs. 4 and 5, dark red boxes) as there is no known high-capacity pumping that would cause such depressions in these areas. Most, if not all, of the anomalous depressions shown in previous figures have been identified in the past through general mapping of the UCCU thickness (Parks, 1990), water-table maps (Konduro-Narsimha, 2007; Ogletree, 2016; Parks, 1990), or groundwater modeling (Jazaei et al., 2018; Villalpando-Vizcaino et al., 2021), with some localized efforts in depressions (A–E) (Figs. 4 (A) and Fig. 5 (A)) that include groundwater tracers, detailed water levels, surface water leakage, and drilling.

Within the MLGW Sheahan wellfield (Fig. 3 and Figs. 4 (A) and 5 (A)), depression (A) exists in the water-table with a 9-m drop 3 km long from Nonconnah Creek north towards the center of the wellfield. This depression has been previously identified and characterized through surface water leakage observations (Larsen et al., 2013; Nyman, 1965), groundwater tracers (Graham & Parks, 1986; Larsen et al., 2003, 2013), water-table depressions (Konduro-Narsimha, 2007; Ogletree, 2016; Parks, 1990), drilling (Hasan personal communication, 2021), and groundwater modeling (Torres-Uribe et al., 2021; Villalpando-Vizcaino et al., 2021).

Depression B, east of the MLGW Allen wellfield, was also noted by Bradshaw (2011) using tracer data as two potential breaches near Cane Creek; however, the exact location was not defined. Additional reports from the Memphis Defense Depot (Memphis Depot; Fig. 3) indicate a connection between the water-table and Memphis aquifers based on geologic cross-sections (HDR, 2017). Depression C has been previously identified near the MLGW Lichterman wellfield which is characterized by thinning or absent UCCU, a downward hydraulic gradient between the water table and Memphis, and areas of inter-aquifer water exchange aquifers (Graham & Parks, 1986; Nyman, 1965; Smith, 2018). Similarly, Depression E was observed by Konduro-Narsimha (2007), Gallo (2015), and Ogletree (2016) near the confluence of two branches of Fletcher Creek, correlating with a suspected breach identified by Parks (1990).

Depression D is located near the former Shelby County Landfill in Shelby Farms (Fig. 3), where Bradley (1991) identified and confirmed a breach directly north of the landfill. Additional studies to substantiate and delineate this breach include seismic reflection (Waldron et al., 2009), electrical resistivity and geochemical analysis (Schoefernacker, 2018), groundwater tracers (Mirecki & Parks, 1994), groundwater modeling and geophysical methods (Gentry et al., 2006), and water-table maps (Konduro-Narsimha, 2007; Ogletree, 2016; Parks, 1990).

Previous water-table maps (Konduro-Narsimha, 2007; Ogletree, 2016; Parks, 1990) identified Depressions A through E; however, the shape and extent differ due to changes in data control and the methodology followed to generate groundwater contours. Although the extent of anomalous depressions are an indicator or a potential breach, they do not provide a detailed shape and orientation due to lack of borehole or well control. The general depressions observed in the water-table maps are somewhat circular as they tend to be centered around a single control point. To better characterize the shape and size of the breaches, the water-table map method should be complemented by additional methods such as detailed geologic mapping, additional boreholes, and geophysical methods.

**Fig. 6:**
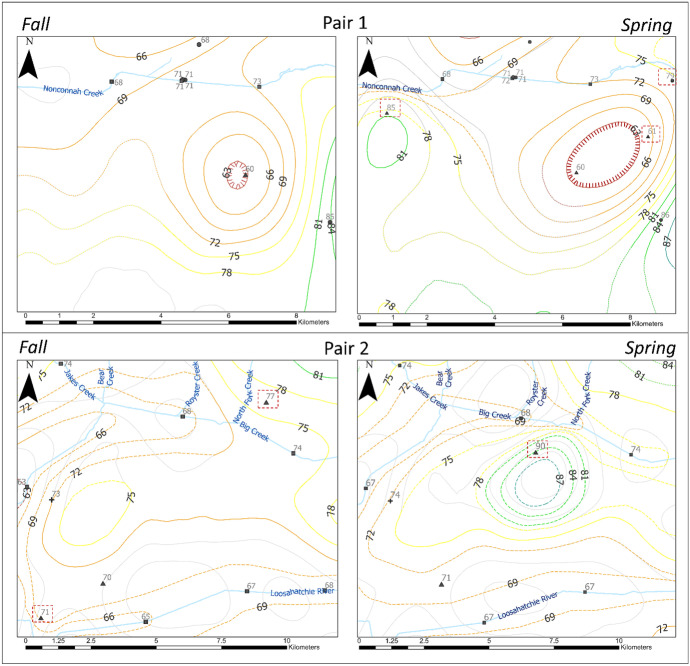
Significant change in contour shape and dimensions from fall to spring following the addition of a single control point in the latter season in each case, indicated in a red dashed box. Each pair (1 and 2) is obtained from the same spatial footprint, all indicated in dark blue dashed boxes in Figs. 4 Fig. 5. Gray lines represent original contours from co-kriging where dashed-colored contours represent manual adjustments proximal to streams. Dotted lines represent approximate locations. Dashed lines were manually modified. Hatched lines represent depressions

When comparing differences in the water-table between fall (Fig. 4) and spring (Fig. 5), the most significant differences are found where data control is inconsistent as also observed by Ogletree (2016). Generally, wells and surface water features were measured during both seasons, but some historical sites had available data for only one of the two seasons, causing data inconsistencies. Figure 6 shows some areas where there is a significant change in the water-table due to data control issues. Each pair (1 and 2) represents the same spatial footprint shown in Figs. 4 and 5.

In Fig. 6, Pair 1, the depression elongates to the northeast as two additional historical points are available for spring. Also, an 81 masl additional peak is observed in spring, south of Nonconnah Creek, due to the addition of a historical point. On the other hand, in Fig. 6, Pair 2, the historical point added in spring produces a new peak that was not observed in the fall surface. This produces a water level rise of 12 m. In areas where control is maintained between the seasons, the general structure of the contours remains very similar, only changing in level, but not in shape. Similarly, areas that heavily relied on topography data for interpolation due to lack of data control remained unchanged in shape and level regardless of the season.

### Seasonal analysis

**Fig. 7:**
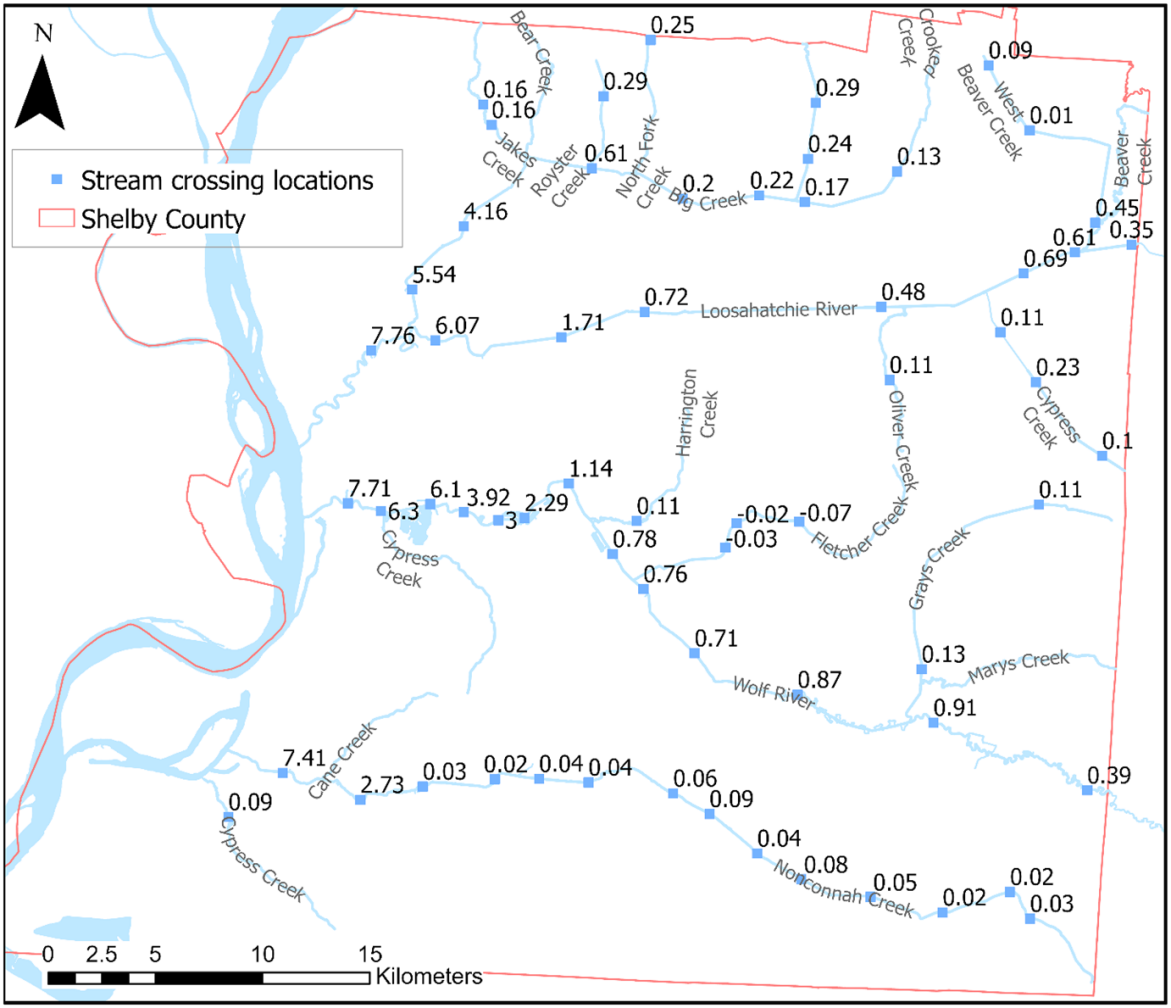
Surface water seasonal change. Squares represent stream-bridge crossings, and numbers represent seasonal change between fall 2020 and spring 2021

**Fig. 8:**
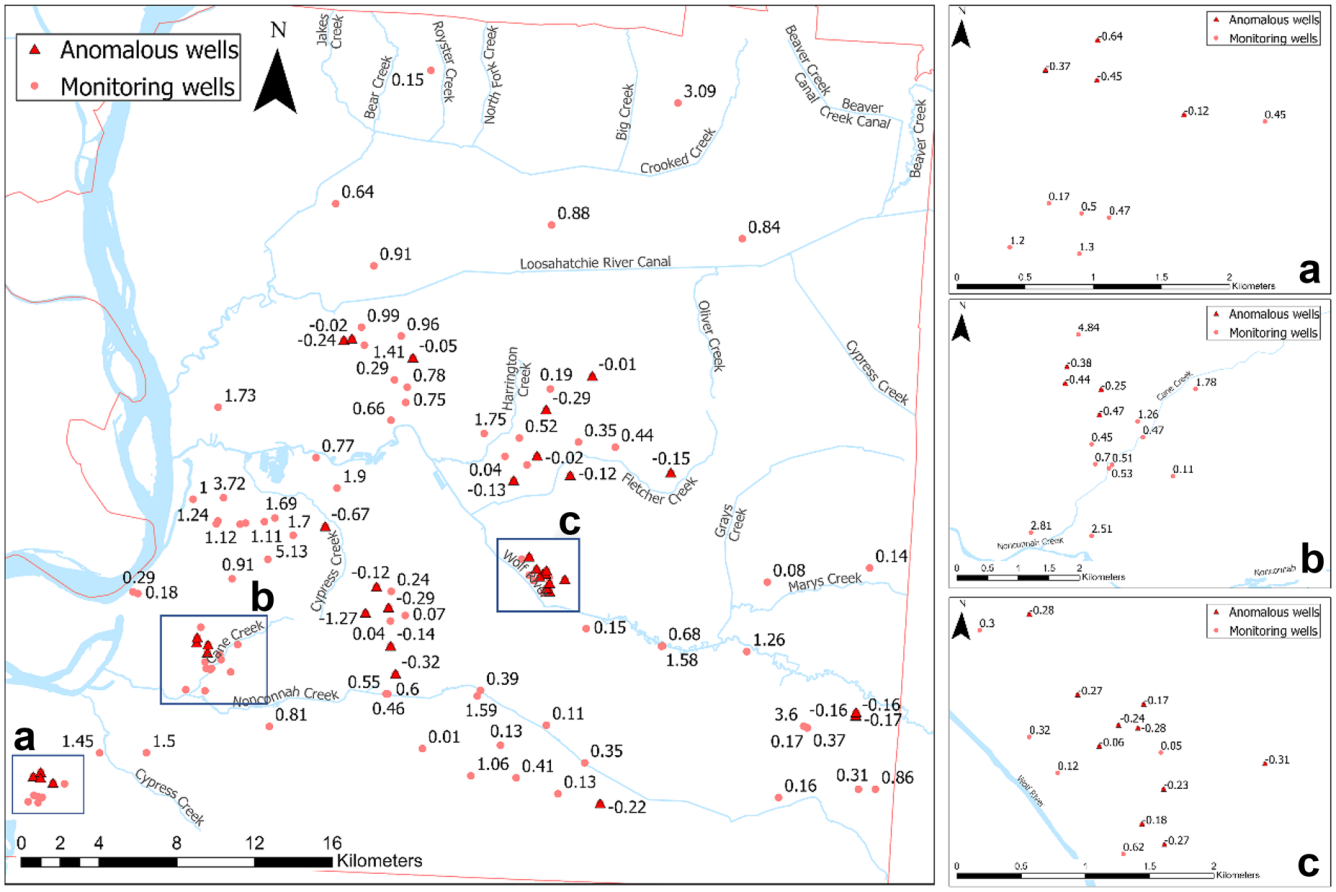
Groundwater seasonal change. Boxes (a–c) are zoomed-in images of clustered features in (a) Davis wellfield; (b) Allen wellfield; and (c) Shelby Farms. Anomalous seasonal changes (higher in fall than in spring) are represented as red triangles and the remaining features (higher in spring than in fall) are shown as pink circles

It was anticipated that spring water levels would be higher than those in the fall since the rainy season is during the winter and spring months. According to the USGS National Water Information System (NWIS) surface water database, the Wolf and Loosahatchie River stages typically remain at baseflow conditions between the months of July and December and rise to 4.5 m on average, reaching their maxima during April and May. Nonconnah Creek’s stage remains more stable throughout the year, only rising after precipitation events but returning to baseflow conditions shortly (a couple of days) after. This behavior was observed in the surface water locations between fall and spring; however, groundwater showed an anomalous seasonal behavior in some locations throughout the county, differing from the expected higher levels in spring than in fall. Figures 7 and 8 show water level changes between fall 2020 and spring 2021 for surface water and groundwater measurements.

Surface water locations near the Mississippi River are affected by backwater conditions that can reach as much as 9 km upstream in the three major rivers in Shelby County. Backwater conditions can cause a rise in water levels during the spring by as much as 7 meters near the confluence of these rivers with the Mississippi River, as seen in Fig. 7. Water level changes between seasons became less significant moving upstream. Generally, surface water levels throughout the county were higher during spring with some exceptions such as Fletcher Creek, a tributary to the Wolf River in the central portion of the county, where water levels were an average 4 cm lower during the spring. Water levels along the rest of the tributaries rose less than 30 cm from fall to spring.

Groundwater levels showed unexpected behavior in some cases, as seen in Fig. 8. Out of 124 groundwater monitoring sites measured during both seasons, 35 had higher elevations during the fall when compared to the spring. This resulted in a negative seasonal change (i.e., spring minus fall), which was considered abnormal. The average negative seasonal water level change was 23.6 cm with a standard deviation of 15.7 cm. The remaining wells behaved as expected, with a positive seasonal change and an average variation of 88.1 cm with a standard deviation of 88.7 cm.

Although the negative seasonal variation was less significant, a preliminary analysis was conducted to relate abnormal water level variations to a physical cause, such as proximity to open water bodies or confirmed breaches. Land use was also considered, assuming that water infiltration is greater among more vegetated areas rather than impervious, developed zones. Results indicated no apparent causality; therefore, other factors were considered.

An analysis was performed using long-term data recorded by pressure transducers (Solinst Inc. Levelogger®) deployed in 12 water-table monitoring wells throughout the county (Fig. 3). These transducers have been collecting pressure data every 15 min from 2019 to 2021. The objective was to observe whether short-term or long-term behaviors of the water-table provided any reasoning for the negative seasonal differences. Short-term variation was set over a 2-week period, both with a rolling average and specific to the survey periods. As provided in Table 3, rolling 2-week averages were taken at each instrumented well to see head variations. Likewise, the average variation of water levels during the survey periods is also listed (i.e., 19 days for the fall survey and 12 days for the spring).

**Table 3:** Rolling average water level 2-week variation. Values are in cm

	Total		Fall (9/14/20–10/2/2020)		Spring (3/29/2021–4/9/2021)	
Well ID	Average	SD	Average	SD	Average	SD
Sh:K-156	15.33	4.90	11.89	1.48	17.82	1.67
Sh:R-032	10.00	4.99	5.97	0.54	12.29	2.70
Sh:J-172	28.71	9.26	27.30	3.28	44.53	14.57
Sh:J-206	25.99	7.67	20.16	2.51	33.93	8.85
Sh:J-196	24.89	6.97	21.86	2.68	34.89	7.79
Sh:J-220	22.23	8.87	15.38	2.54	32.75	6.88
Sh:K-171	36.28	19.19	17.61	5.45	57.14	13.77
Sh:L-110 UR-25S	78.84	75.26	19.02	10.01	142.56	64.98
Sh:K-169	28.44	9.66	21.81	3.95	46.52	12.83
Sh:K-163	39.99	15.56	44.76	19.20	38.56	6.10
Sh:J-242	62.35	47.10	17.81	5.16	102.39	48.65
GG-MW1	27.77	27.94	5.81	2.27	110.30	14.99
Average	33.40	19.78	19.11	4.92	56.14	16.98

A total average of 33.40 cm with a standard deviation (SD) of 19.78 cm were obtained by averaging the complete data set (2019–2021) of collected data from all 12 wells. For fall and spring, 19.11 cm (SD of 4.92) and 56.14 cm (SD of 16.98) averages were obtained, respectively. This suggests water levels tend to be less variable during fall than during spring, likely due to recharge in the spring.

During the data collection period, it was planned that all wells in clustered areas (e.g., MLGW well fields, Shelby Farms) were surveyed on the same day to ensure better data consistency between neighboring wells. However, given the size of the county and the distribution of monitoring points, each survey took between 2 and 3 weeks to complete. Within this period, it is apparent in Table 3 that water levels could have shifted ±33.40 cm (e.g., using the total average), depending on the day collected.

**Fig. 9:**
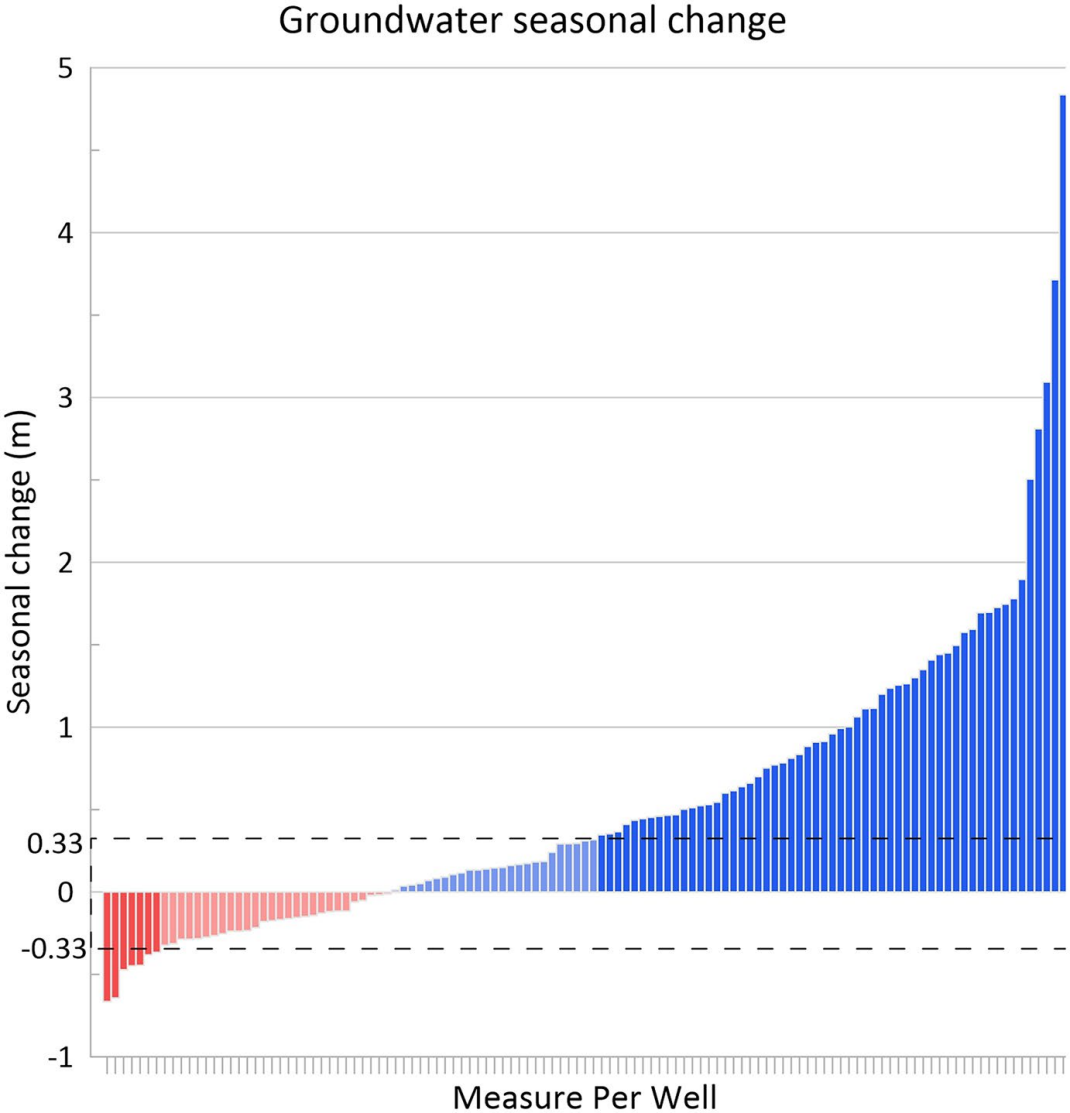
Seasonal change in all wells measured for both seasons. The black dashed box represents the total average ±33.40 variation attributed to short-term fluctuations of the water-table aquifer. Bars falling within the box (80% of red, 30.5% of blue) are shown as a lighter color. Additional IDs and water elevations for each well are found in Appendix 1 Table 7

Figure 9 shows the seasonal change observed for every measured well. The dashed black box represents the short-term total average ±33.40 cm variation of the water-table aquifer as presented in Table 3. Any seasonal change within the black box is attributed to a short-term variation of the water-table, rather than a seasonal effect on the aquifer.

Out of all the water levels that decreased from fall to spring (i.e., negative change or red bars), 80% fall within the black box. Conversely, only 30.5% of the blue bars (i.e., higher levels in the spring compared to fall) fall within the box. This further shows that seasonal change was more significant in wells with higher levels during spring, but still does not fully explain the lower readings beyond the −0.33 m threshold for some wells during the spring as compared to fall.

**Fig. 10:**
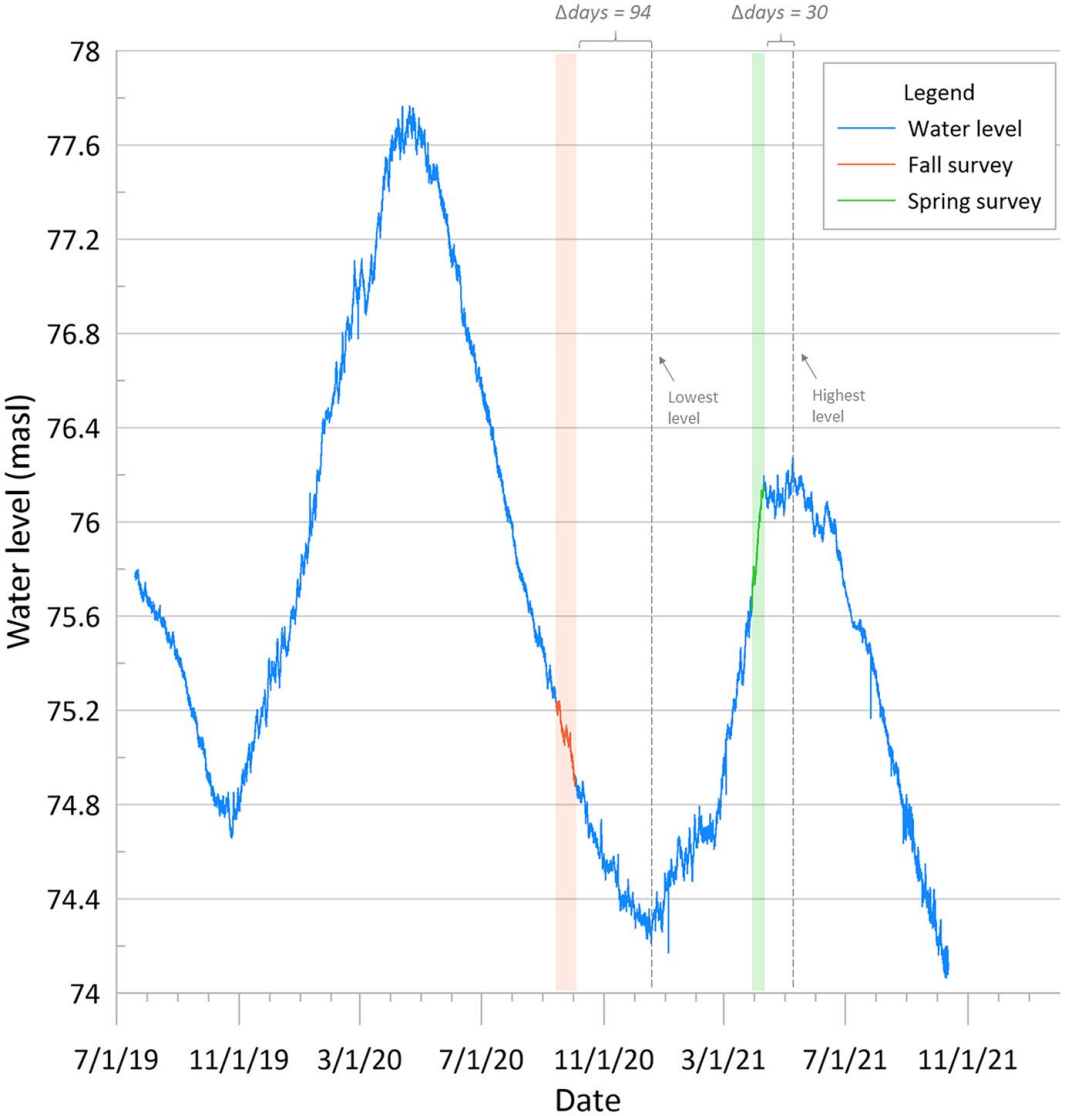
Continuous groundwater elevation for well Sh:J-172. Data collection periods for fall (orange shade) and spring (green shade) are also shown, along with the lowest and highest (i.e., dry season and wet season) water levels recorded for each season, displayed as gray dashed lines

**Fig. 11:**
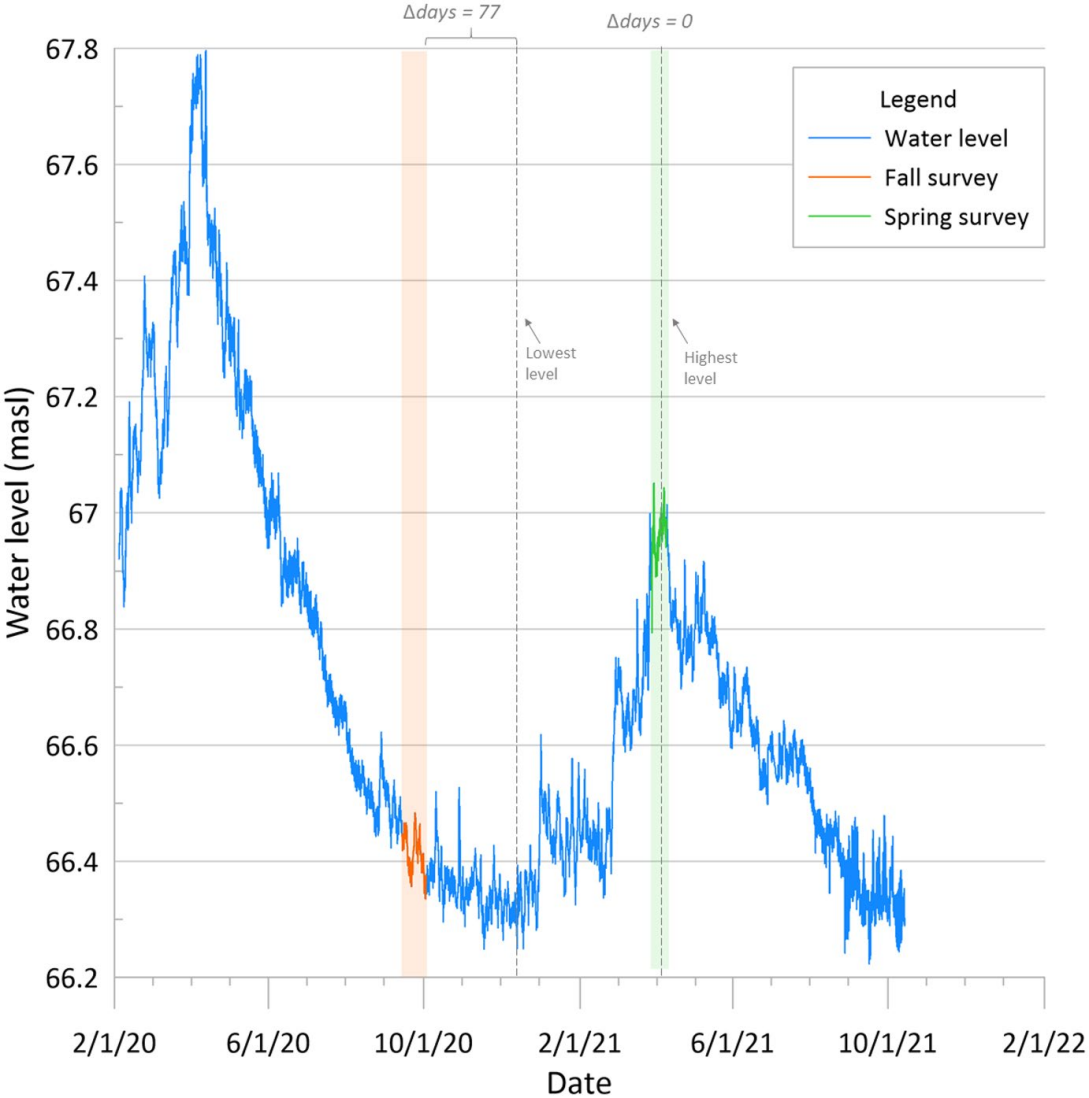
Continuous groundwater elevation for well Sh:J-220. Data collection periods for fall (orange shade) and spring (green shade) are also shown, along with the lowest and highest (i.e., dry season and wet season) water levels recorded for each season, displayed in gray dashed lines

Following the seasonal change analysis for all wells, the long-term variations (July 2019 to October 2021) of the water-table aquifer were analyzed using the observed readings collected by pressure transducers. Wells Sh:J-172 and Sh:J-220 (Figs. 10 and 11) were selected to illustrate the range of water-level fluctuations seen in the wells listed in Table 3.

With longer periods of record, seasonal groundwater patterns become more apparent. In the case of well Sh:J-172, data were collected 94 days earlier than the lowest value it had for fall, and 30 days earlier than the highest level in spring. Similarly, for well Sh:J-220, data collection for fall occurred 77 days before the water reached its lowest point, while spring data were collected right as the water-table approached its highest peak of the year. A similar analysis was conducted with the rest of the wells with transducer data (Table 4). Wells Sh:K-156, Sh:K-163, and Sh:L-110 UR-25S are excluded from the analysis given that Sh:K-156 and Sh:K-163 are located within the breach in Sheahan wellfield, and Sh:L-110 UR-25S is strongly influenced by Nonconnah Creek; hence, resulting in behaviors differing notably from the ones seen in Fig. 10 and Fig. 11. Wells GG-MW1 and Sh:J-242 are also located in close proximity to the Wolf River and Nonconnah Creek, respectively; however, these follow a similar pattern to the rest of the wells in the analysis and, therefore, are included in the analysis. The reason why some wells near the same stream (i.e., L-110 UR-25S and Sh:J-242) have differing water level behaviors requires further investigation and is not addressed in this study.

**Table 4:** Dates of lowest and highest (i.e., fall and spring) water levels were recorded during the data collection periods in comparison to the lowest and highest dates and levels of each season

	Fall 2020						Spring 2021					
	Lowest point		Data collection period (9/14/20–10/2/20)				Highest point		Data collection period (3/29/21–4/9/21)			
Well	Date	Level (masl)	Lowest date	Level (masl)	Days diff.	Level diff.	Date	Level (masl)	Highest date	Level (masl)	Days diff.	Level diff.
R-032	12/28/2020	84.93	10/1/2020	84.97	88	0.04	5/27/2021	85.16	4/7/2021	85.13	50	0.03
J-172	1/4/2021	74.51	10/2/2020	74.88	94	0.37	5/9/2021	76.24	4/9/2021	76.16	30	0.08
J-206	1/28/2021	63.1	10/2/2020	63.29	118	0.19	5/28/2021	63.88	4/9/2021	63.73	49	0.15
J-196	12/29/2020	67.96	10/2/2020	68.22	88	0.26	5/28/2021	68.84	4/9/2021	68.75	49	0.09
J-220	12/18/2020	66.25	10/2/2020	66.33	77	0.08	3/31/2021	67.05	3/31/2021	67.05	0	0
K-171	12/13/2020	76.85	10/2/2020	76.94	72	0.09	4/2/2021	78.79	4/2/2021	78.79	0	0
K-169	11/17/2020	84.47	10/2/2020	84.67	46	0.2	5/9/2021	86.62	4/9/2021	86.56	30	0.06
SAA-1	10/26/2020	62.3	10/2/2020	62.36	24	0.06	3/31/2021	65.48	3/31/2021	65.48	0	0
GG-MW1	12/13/2020	74.04	10/2/2020	74.15	72	0.11	4/2/2021	75.98	4/2/2021	75.98	0	0
Average					75.44	0.16					23.11	0.05

Table 4 lists each transducer water level for the dry and wet seasons, in comparison to the water levels obtained during the water level surveys.

Results indicate that for fall 2020, the water level survey concluded approximately 75 days before the water table reached its lowest level. At the lowest level, water levels were on average 16 cm lower than they were during the last day of the water level survey on October 2^nd^. Considering this 75-day difference, the ideal date to obtain the lowest water levels for fall is around the third week of December. During Spring 2021, four wells were measured when the water-table was at its highest level; however, the remainder were surveyed approximately 23 days too early. It is not a simple case of shifting future spring survey dates as pushing the date forward would capture higher levels in some wells (e.g., Sh:R-032, Sh:J-172, Sh:J-206, Sh:J-196, Sh:K-169) while resulting in lower levels in others (e.g., Sh:J-220, Sh:K-171, Sh:J-242, GG-MW1). Nevertheless, with only an average 5-cm water level difference if shifted by 23 days, data collection at any time during April is appropriate. Considering that water levels fell an average of 16 cm from the last day of the fall survey and rose an average of 5 cm after the last day of the spring surveying if the water-level surveys had been conducted at the right time for both seasons (i.e., lowest and highest levels of the year), water level variations would have been an average 21 cm higher (sum of both seasons shift averages). Recalling the average 23.6-cm seasonal variation within the wells that had higher levels in fall, the abnormal negative seasonal behavior observed may be attributed to the incorrect timing of the water-level surveys.

Given that these ideal dates for data collection are based on data from the past 2 years, an extended period of data was required to identify dates of minimum and maximum levels from the water-table aquifer. There are several wells in Shelby County, screened within the water-table aquifer, with long-term monitoring data. However, most have measurements that are sporadic throughout the year, so it is difficult to estimate the actual dates of the lowest and highest levels from these data. For this reason, an analysis was conducted using well Sh:P-099, which has been monitored daily by the USGS since 1994. Table 5 shows the dates of the highest and lowest level in the aquifer each year since 2015.

**Table 5:** The highest and lowest levels per year recorded in well P-099 from 2015 to 2020

Year	Maximum level	Minimum level
2015	April 26th	November 14th
2016	May 4th	November 19th
2017	May 4th	December 16th
2018	April 28th	December 12th
2019	April 18th	October 7th
2020	April 3rd	November 17th

Based on the dates from Tables 4 and 5, it is observed that the approximate months to capture the lowest and highest levels in the water-table for future monitoring efforts are November and December and April and May, respectively.

### Decadal analysis

**Fig. 12:**
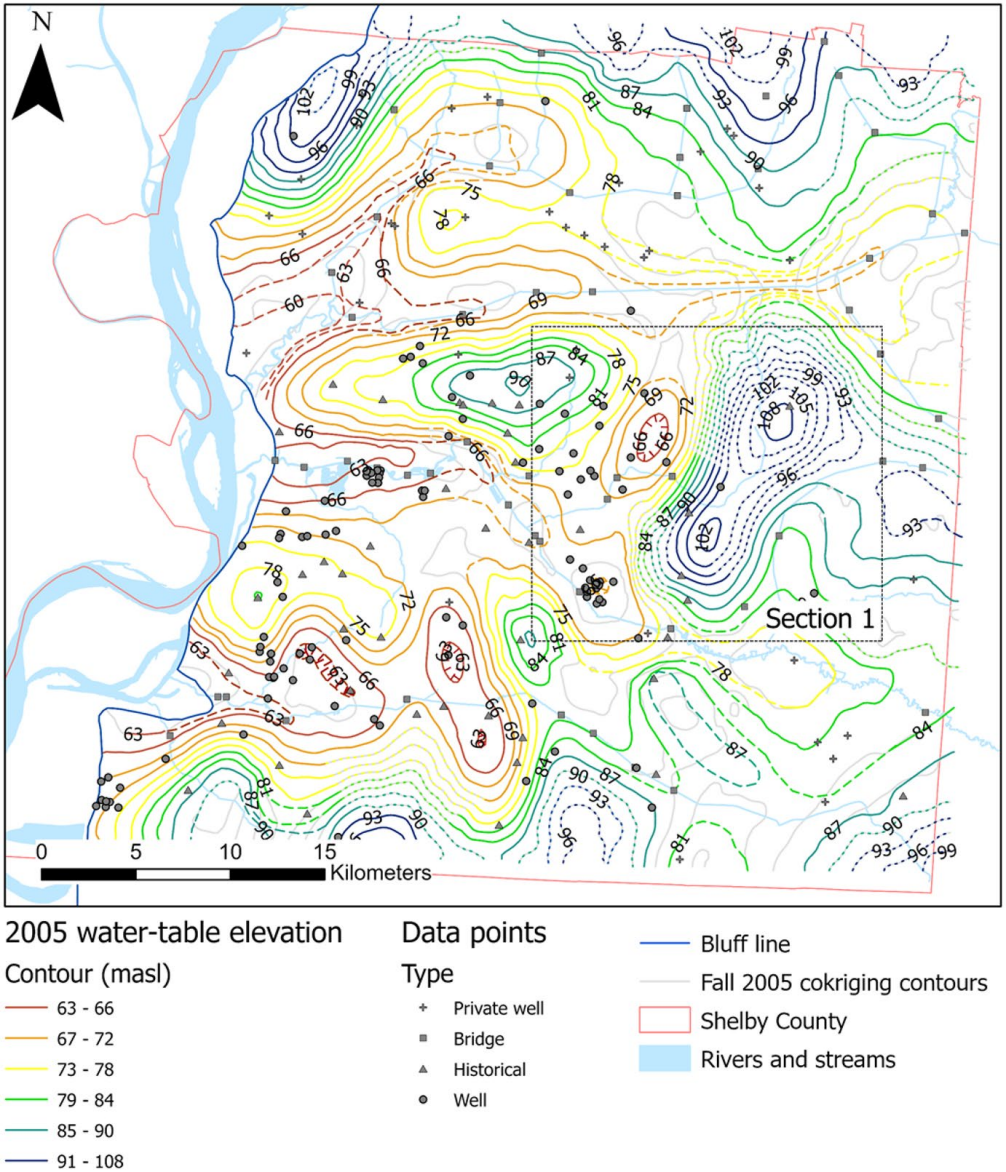
2005 water-table map produced following the same methodology as Figs. 4 and 5, using data collected during fall 2005. Gray lines represent original contours from co-kriging, whereas dashed-colored contours represent manual adjustments proximal to streams. Dotted lines represent approximate locations. Dashed lines were manually modified. Hatched lines represent depressions. Black dotted box represents the section used for decadal comparison and is also seen in Figs. 4 and 13.

**Fig. 13:**
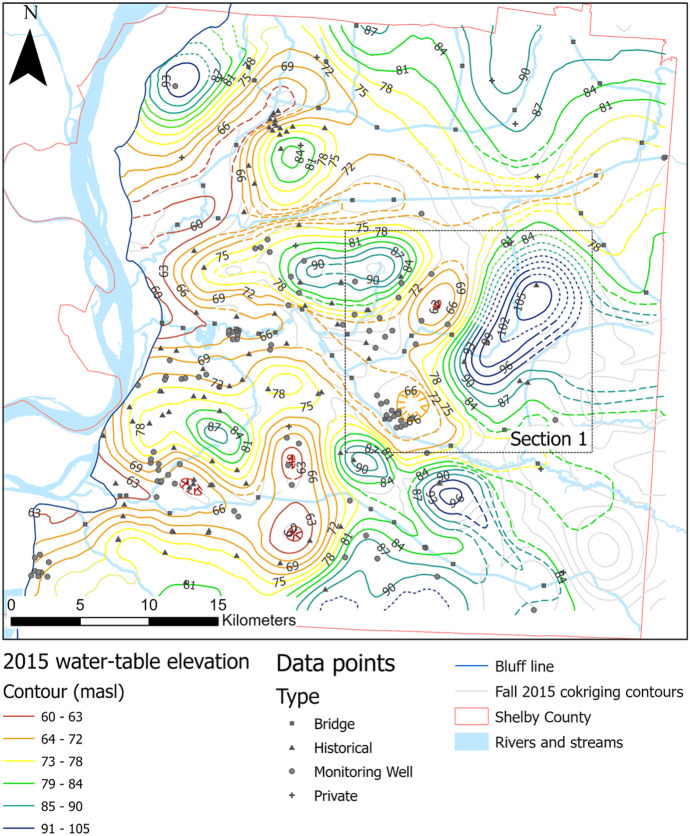
2015 water-table map produced following the same methodology as Figs.4 and 5, using data collected during fall 2015. Gray lines represent original contours from co-kriging, whereas dashed-colored contours represent manual adjustments proximal to streams. Dotted lines represent approximate locations. Dashed lines were manually modified. Hatched lines represent depressions. Black dotted box represents the section used for decadal comparison and is also seen in Figs. 4 and 12

Data collected during the 2005 (Konduro-Narsimha, 2007) and 2015 (Ogletree, 2016) water-level surveys were reprocessed following the same methodology employed for this study to compare water tables over the past 15 years (2005 to 2020; see Figs. 12 and 13). Parks (1990) was not included because his data collection methods differed significantly from later surveys. Data collection for 2005 and 2015 was conducted during the fall months; thus, only the fall 2020 surface was used for comparison purposes. There is significant variability in control for each year, especially with historical data and private wells. Historical sites are problematic since they are temporal and can change as new sites are added and some are closed over time, while private wells are impacted by factors such as well destruction, upgrades that limit port accessibility, owner changes, and denied access. Impacts on these sites affect the overall county control as these locations help fill out the data gaps outside wellfield clusters. Table 6 shows the amount of control points by category and by year.

**Table 6:** Data control for survey years 2005 (Konduro-Narsimha, 2007), 2015 (Ogletree, 2016), and 2020. Numbers in parentheses represent points that match with those measured in 2020

	2005	2015	2020
Public monitoring wells	114 (70)	104 (74)	99
Surface water crossings	56 (50)	52 (51)	69
Private wells	37 (9)	11 (7)	12
Historical sites	42 (0)	99 (5)	19
Total	249 (129)	266 (137)	199

As seen in Table 6, data control generally decreased over time. Public monitoring wells, historical sites, and private well measurements decreased from 2005 to 2020 with only surface water crossings increasing with time. It can be seen that data control increased from 2005 to 2015 but decreased from 2015 to 2020, with 2020 having the lowest data control between the three water-level surveys.

**Fig. 14:**
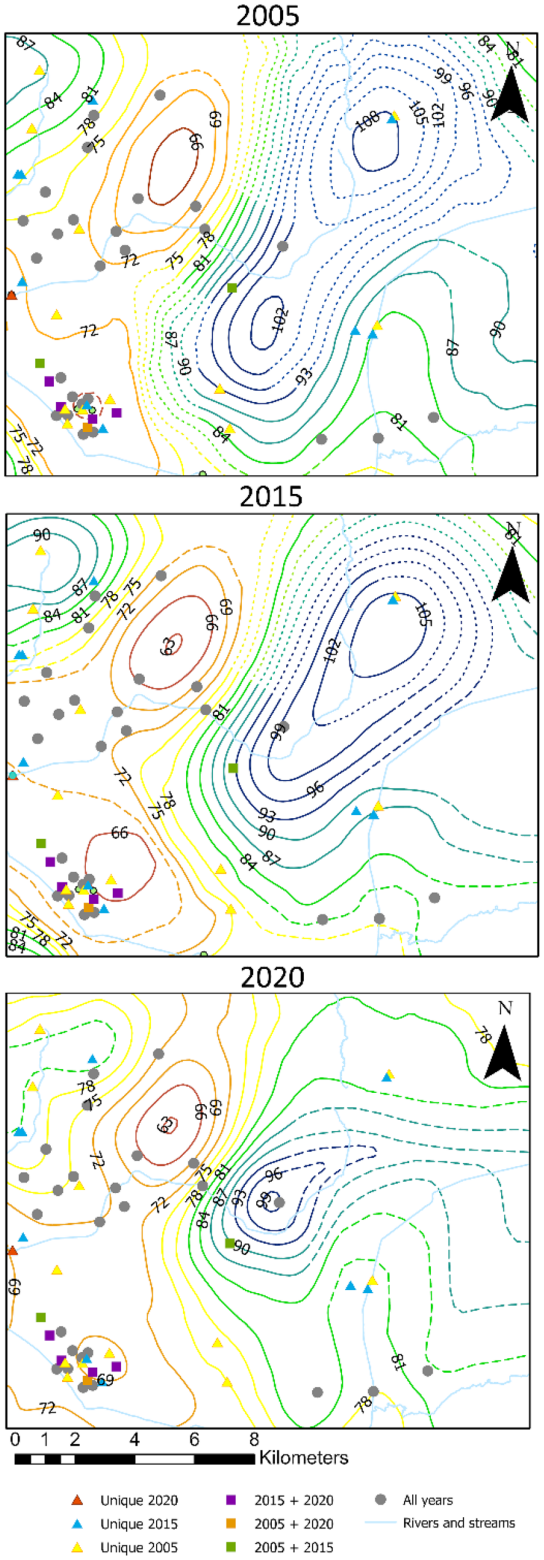
Decadal comparison between water-table maps. Sections have coincident footprints in each year’s map. Contour intervals remain consistent for comparison purposes, except for the highest and lowest values in each case. Surveyed features unique to a single survey are shown in primary-colored triangles; features found in any two survey combinations are shown in secondary color squares; and features for found in all three surveys are displayed as gray circles

The most notable differences between the 2005 and 2015 water-table surfaces and the 2020 are found in areas with significant changes in data control. Ogletree (2016) analyzed the differences in water-table elevations in relation to data control changes between 2005 and 2015 and concluded that considerable changes between water surfaces are not the result of physical changes in water-level elevations but differences related to data control. Similar conditions are observed when comparing with 2020 data. Figure 14 shows Section 1 (Figs. 12 and 13) a clear example of where significant changes in contour shape and levels can be attributed to differences in data control.

For Section 1, the size of the observed elevated water level was significantly reduced from 2005 and 2015 to 2020. Both in 2005 and 2015, there was a historical site control point slightly offset from each other (yellow and blue triangles) that created a higher water level to the northeast of Section 1. In 2020, these historical points were absent, centralizing the peak around a single point towards the center of the map. The highest contour elevation was also reduced from 108 m in 2005 to 99 m in 2020. Areas of the county with less significant changes in data control have less variation in the shape of contours, particularly within the MLGW wellfields where data control tends to be more clustered and consistent.

**Fig. 15:**
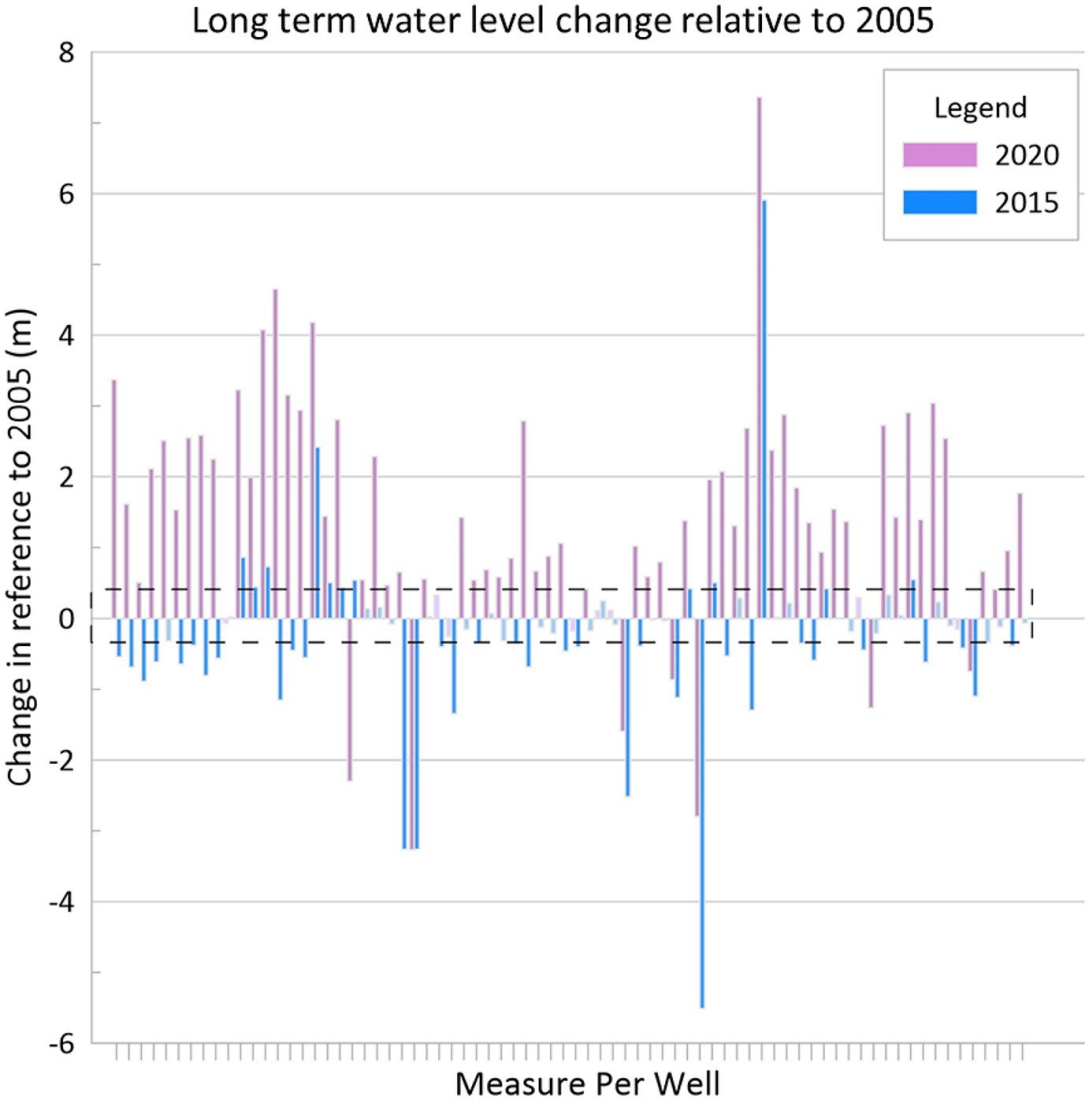
Long-term water level change. Values are obtained by subtracting the 2005 water level from 2015 and 2020 levels. Negative values indicate lower levels in these years than in 2005, while positive values indicate higher levels. The black dashed box represents the total average ±33.40 cm short-term variation of the water table. Number IDs for each well are found in Appendix 1 table 7

An additional analysis was conducted to observe the long-term water-level variation from 2005 to 2015 and 2020 (Fig. 15).

Results indicate that in 2015, water levels were generally lower than in 2005, while water levels in 2020 were overall higher than in 2005, with some exceptions in both cases. For illustrative purposes only, the black dashed box represents the average ±33.40 cm variation that could be attributed to short-term water-level fluctuations (Table 3) rather than a long-term change—recall that the short-term variation was over a shorter period, 2019–2021. On average, water levels were 0.27 m lower in 2015 than in 2005, while water levels in 2020 were 1.33 m higher than in 2005.

**Fig. 16:**
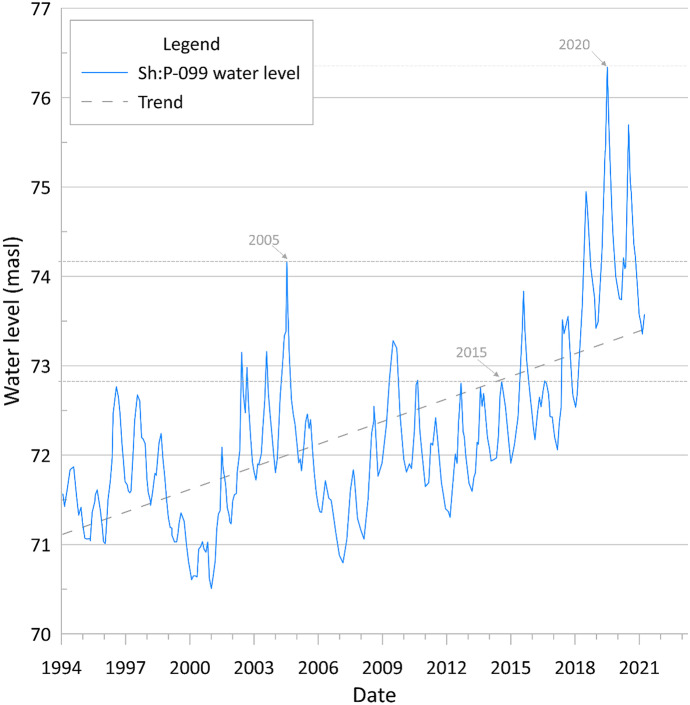
Well Sh:P-099 historical groundwater level (MASL) obtained from long-term USGS monitoring records. Water-level survey years are indicated with arrows and the highest level for each survey is represented in gray dashed lines for comparison purposes

To further examine water-table fluctuation trends observed for each year, a comparison with long-term monitoring data from well Sh:P-099 was conducted. This well is near the Memphis Zoo (see Fig. 3) and has several water features that may leak and provide artificial recharge to the water-table aquifer. However, assuming the leakage is near constant, the general trend of the water-table is relatively affected and simply shifts up. According to this site, which is the only continuously recording monitoring well for the water-table aquifer in the county, groundwater levels in the water-table aquifer have been rising since monitoring began in 1994. Although in 2015, water levels fell below 2005 and 2020 levels (Fig. 16), matching the behavior shown in Fig. 15 for Number ID 48.

According to the National Oceanic and Atmospheric Administration (NOAA), precipitation in Shelby County has increased on average by 190 mm over the last 30 years (NOAA, 2021), which might account for the rising trend in water levels observed in well P-099. However, records show that 2005 was a drier year with an annual precipitation of 1084 mm, lower than 2015 and 2020 with 1403 and 1441 mm, respectively. This suggests that water levels in the water-table aquifer are influenced by more than just recharge from precipitation, or by differences in time scales between rain events and aquifer fluctuations.

### Probability surface

**Fig. 17:**
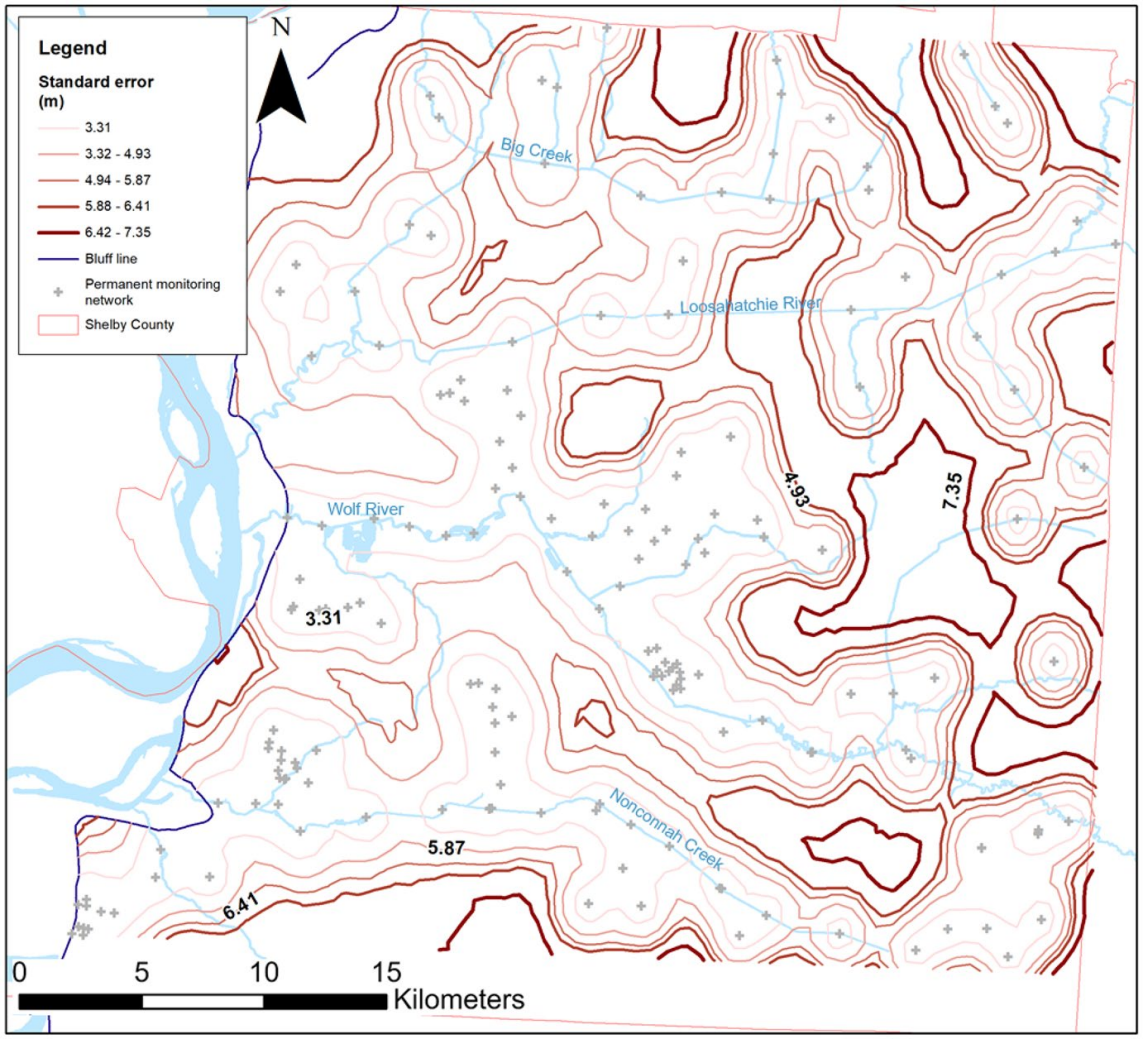
Standard prediction error map. Bold, darker red contours represent a higher error, usually located in areas with little to no control. The permanent monitoring network including public and private wells and stream crossings is shown in light gray crosses

The last analysis performed using the water level survey data was to ascertain areas of high prediction error in the derived surfaces which would direct future efforts to fill those gaps before the next survey. A map showing the areas with the highest prediction error was generated (Fig. 17) based on survey location and the standard deviation of the values obtained from the interpolation.

A total of 179 permanent monitoring locations from the monitoring network, which includes public and private wells and stream crossings, were used for this analysis. Due to the changing nature of historical sites, they were removed from this analysis. Significant errors (data gaps) ranging from 5.88 to 7.35 m are shown between the major rivers (i.e., Loosahatchie River, Wolf River, and Nonconnah Creek) in eastern Shelby County, where the highest error area of 7.35 m is found as it is away from utility wellfields, accessible private wells, and between river crossings. Additionally, there are pockets of high error along the periphery of the county as well as along the Loosahatchie River and northward where the majority of control is only surface water. Northern Shelby County monitoring control relies almost solely on stream crossings, and if the analysis was done exclusively considering monitoring wells, this area would have a significantly higher error than the one observed in Fig. 17.

## Conclusion

Mapping water levels of a water-table aquifer is useful for identifying areas of preferential leakage to the underlying confined aquifer through potential breaches in the intervening aquitard. Water-table maps were generated for fall 2020 and spring 2021, successfully meeting aim one of the research. These maps show previously observed groundwater depressions within Sheahan wellfield, Shelby Farms, in areas west of Lichterman wellfield, east of Allen wellfield, and east of McCord wellfield, which indicate potential aquitard breaches, which addresses the second objective of the research. Though no new anomalous water-table depressions were identified in this investigation, it was observed, as in Ogletree (2016), that loss in control (primarily from loss in private wells and historical measures) greatly impacts our ability to gain needed detail in the water-table surface in prior mapped areas. Outside of these historical depressions, no new depressions were observed with the current monitoring network.

This investigation was the first time a water-table survey was conducted in two seasons: fall (dry) and spring (wet), where past surveys including Parks (1990) were performed in the fall. Changes between seasons were observed, and by observing continuous monitoring of water levels in the water-table aquifer, it was possible to identify more appropriate times to conduct future surveys in the fall season (local minimum) and spring (local maximum). Regarding aim three, typical seasonal behavior is observed in surface water bodies (i.e., rivers and tributaries) as water levels were generally higher during spring than fall, more significantly near the confluence of the three major rivers in the county with the Mississippi River where values were as high as 7 m. Seasonal differences decreased as locations moved upstream with some exceptions attributed to river geomorphology. Anomalous seasonal behavior (i.e., higher water levels during fall than spring) is seen in 35 out of the 124 wells surveyed for both seasons, with an average seasonal variation of 23.6 cm. The remaining wells showed an average 88.1 cm rise in water levels from fall to spring. A short-term analysis of the water-table based on pressure transducer data showed an average ±33.40 cm 2-week variation in the water-table aquifer. Additional pressure transducer observations showed that the fall water-level survey concluded approximately 75 days before the water-table approached its lowest level in fall (16 cm lower) while the spring survey occurred 23 days before it reached its highest peak (5 cm higher) in spring. Hence, abnormal seasonal change is attributed more to the timing of the water-level surveys rather than a physical phenomenon of hydrogeology.

Past investigations into developing a representative surface of the water-table followed differing approaches. In this study, a consistent methodology was employed for the surfaces of fall 2020 and spring 2021 but was reapplied to the data collected in 2015 and 2005. By applying a consistent methodology against past investigations, comparative analyses between the years were improved, whereby any inconsistencies arising from inconsistent methodologies were eliminated. From decadal data, it was observed that groundwater levels were higher in 2020 than in 2015 and 2005, while levels were lower in 2015 than in 2005. Differences in the water-table maps for each survey are attributed to significant differences in data control. To identify areas to potentially improve the monitoring network in the future, a standard error map was generated and showed a prediction error of up to 7.35 m in areas with no control, more significantly in eastern Shelby County and between the major rivers, with the northern part of the county relying mostly on river crossing data. It goes without saying that improved control through additional observation points would greatly improve the detail of the water table surface. Inclusion of more private wells through a stronger relationship with the Shelby County Health Department, which permits those wells and samples them annually for water quality, would be of huge benefit, although it is expected that many private wells extend into the deeper Memphis aquifer and may not withdraw from the water-table aquifer. Possibly, geophysical techniques that produce a notable signal change at the point of full saturation (i.e., water-table surface) could provide a means of obtaining water levels in a non-invasive manner (i.e., not via drilling). Certainly, drilling new observation wells in areas that would provide much-needed control via the identified prediction error zones would be beneficial and could be instrumented with real-time monitoring which proved useful when identifying local minimum/maximum water levels. Lastly, the inclusion of more surface water monitoring stations in the tributaries to the mainstream systems would improve the depiction of groundwater gradients in the hill-valley sections as the water-table tends to conform to topography, and less reliance on topography via cokriging to define the surface would add to the surface validation.

## Data Availability

The datasets generated during and/or analyzed during the current study are available from the corresponding authors upon reasonable request.

## Appendix 1

**Table 7:** Water levels from fall 2005, fall 2015, fall 2020, and spring 2021 surveys

Number ID	USGS ID	Owner ID	Lat	Long	Water-table elevations in meters above mean sea level (masl)			
					Spring 2021	Fall 2020	Fall 2015	Fall 2005
1	Sh:H-022	4T7	35.02	−90.13	74.16	73.99	70.08	70.62
2	Sh:H-023	4T9	35.03	−90.13	69.4	69.76	67.46	68.15
3	Sh:H-024	4T6	35.02	−90.13	70.53	69.33	67.93	68.82
4	Sh:J-171	1T2	35.09	−90.03	66.69	66.16	65.27	64.55
5	Sh:J-172	4T1	35.02	−90.12	75.67	75.2	72.48	73.09
6	Sh:J-173	1T1	35.1	−90.04	75.95	76.39	73.57	73.88
7	Sh:J-174	1T3	35.09	−90.03	66.65	65.95	64.94	63.91
8	Sh:J-188	4T5	35.02	−90.13	74.99	73.69	71.52	72.16
9	Sh:J-189	4T4	35.02	−90.13	76.01	75.51	72.58	72.96
10	Sh:J-190	4T8	35.03	−90.12	71.02	71.47		
11	Sh:J-191	4T10	35.03	−90.12	70.2	70.84	67.45	68.25
12	Sh:J-194	4T3	35.03	−90.12	75.02	75.14	72.33	72.89
13	Sh:J-195	1T7	35.08	−90.03	65.83	63.32	63.41	63.39
14	Sh:J-196	1T6	35.08	−90.02	68.45	68.34	65.98	65.12
15	Sh:J-197	1T10	35.09	−90.03	69.24	68.77	67.23	66.78
16	Sh:J-198	1T12	35.09	−90.03	70.71	70.26		
17	Sh:J-200	1T11	35.09	−90.03	72.56	73.04	69.69	68.96
18	Sh:J-201	1T8	35.09	−90.03	72.75	71.48		
19	Sh:J-202	1T9	35.1	−90.03	75.39	75.64	69.83	70.98
20	Sh:J-203	1T4	35.1	−90.04	76.86	77.24	73.63	74.08
21	Sh:J-205	1T14	35.11	−90.04	84.16	79.33	75.84	76.39
22	Sh:J-206	1T5	35.1	−90.02	63.37	61.59	59.82	57.41
23	Sh:J-215 UR-1		35.03	−90.11	75.35	74.91		
24	Sh:J-216 UR-2		35.05	−90.07	78.08	76.58		
25	Sh:J-217 UR-3		35.04	−90.09	69.76	68.31		67.38
26	Sh:J-220	1T13	35.09	−90.03	66.89	66.38	65.45	64.94
27	Sh:J-242	SAA-1	35.08	−90.04	65.32	62.51		
28	Sh:K-075	0T52	35.09	−89.93	67.29	67.61	65.24	64.80
29	Sh:K-137	0T51	35.12	−89.93	65.07	65.35	68.19	67.65
30	Sh:K-155	0T55	35.11	−89.92	64.33	64.26	63.85	63.71
31	Sh:K-156	MLGW-96S, 0T56	35.1	−89.93	59.6	59.74	57.61	57.45
32	Sh:K-163	MLGW-99S, 0T59	35.11	−89.93	55.19	55.15		
33	Sh:K-169 UR-11		35.04	−89.88	86.02	84.95	84.40	84.48
34	Sh:K-171 UR-13S		35.08	−89.88	78.73	77.14	73.22	76.48
35	Sh:L-094	3T1	35.04	−89.86	87.77	87.36	87.37	90.63
36	Sh:L-096	3T2	35.06	−89.87	91.32	91.19	90.66	90.63
37	Sh:L-108 UR-23		35.03	−89.81	92.86	93.08	92.35	92.95
38	Sh:L-110 UR-25S		35.05	−89.82	82.56	82.21	82.37	82.44
39	Sh:L-113 UR-27		35.11	−89.82	73.05	72.9	72.16	72.56
40	Sh:M-044	D-1	35.08	−89.67	84.11	84.27		
41	Sh:M-059	PR-HPCH	35.04	−89.71	84.13	83.97	82.88	84.22
42	Sh:M-062	STGEO-N	35.1	−89.73	78.2	76.94	75.36	75.52
43	Sh:O-229	0T2	35.15	−90.02	73.63	72.52	71.63	71.97
44	Sh:O-244	0T1	35.15	−90.03	73.61	72.26	71.65	71.57
45	Sh:O-245	0T3	35.15	−90.01	73.43	72.32	71.42	71.73
46	Sh:O-252	0T4	35.15	−90.03	73.26	72.02	73.74	73.67
47	Sh:O-254	0T5	35.16	−90	74.8	73.36	72.15	72.51
48	Sh:P-099	5T1	35.15	−89.99	76.38	74.68	71.21	71.90
49	Sh:P-107	PR-CL-EG	35.24	−89.93	82.06	81.1	80.31	80.43
50	Sh:P-197	6T1	35.24	−89.96	78.59	78.61	77.53	77.73
51	Sh:P-210	0T53	35.13	−89.94	64.06	64.18		
52	Sh:P-211	0T54	35.13	−89.94		62.32		
53	Sh:P-212	0T6	35.16	−90	74.86	73.17	71.65	72.11
54	Sh:P-213	6T2	35.24	−89.95	74.96	73.55	73.33	73.73
55	Sh:P-215	6T3	35.24	−89.96	78.16	78.4	77.81	77.98
56	Sh:P-216	6T4	35.25	−89.95	79.24	78.25	78.39	78.14
57	Sh:P-217	0T57	35.13	−89.93	66.8	66.56		
58	Sh:P-220 UR-4		35.2	−89.93	68.2	67.54	67.33	67.42
59	Sh:P-221 UR-5		35.22	−89.93	89.96	89.67	88.74	91.26
60	Sh:P-222 UR-6		35.23	−89.92	89.2	89.26	87.85	88.24
61	Sh:P-223 UR-7		35.21	−89.93	81.02	80.27	79.66	79.68
62	Sh:P-224 UR-8		35.2	−89.88	82.88	81.13	80.31	80.34
63	Sh:P-226 UR-10		35.19	−89.91	68.31	68.31		
64	Sh:P-255	0T7	35.17	−90.03	74.79	71.08	70.83	71.94
65	Sh:Q-094 UR-19	2T1	35.19	−89.86	75.3	75.2	74.25	73.82
66	Sh:Q-102		35.13	−89.83	68.07	68.39	66.28	71.79
67	Sh:Q-110		35.14	−89.86	70.88	70.58	69.13	68.62
68	Sh:Q-111		35.14	−89.85	70.41	70.7	68.10	68.62
69	Sh:Q-112		35.14	−89.85	70.18	69.86	68.84	68.55
70	Sh:Q-128		35.14	−89.84	68.22	68.39	64.41	65.71
71	Sh:Q-129		35.14	−89.84	68.15			
72	Sh:Q-134		35.13	−89.85	69.8	69.68	68.23	62.32
73	Sh:Q-135		35.13	−89.84	69.01	69.27	66.92	66.90
74	Sh:Q-138		35.13	−89.85	67.98	68.04	65.38	65.16
75	Sh:Q-140		35.13	−89.84	67.38	67.33		
76	Sh:Q-141		35.13	−89.84	68.64	68.87	66.68	67.03
77	Sh:Q-142		35.13	−89.84	70.69	70.07	68.13	68.72
78	Sh:Q-143		35.13	−89.84	69.05	69.23		
79	Sh:Q-156	2T5	35.19	−89.85	74.93	74.95		
80	Sh:Q-157 UR-15	2T2	35.2	−89.86	79.55	79.03	78.52	78.10
81	Sh:Q-158	2T4	35.18	−89.86	72.76	72.88	71.35	71.34
82	Sh:Q-159	2T3	35.19	−89.87	77.43	77.38	75.84	76.02
83	Sh:Q-161 UR-16		35.21	−89.85	72.85	73.13	72.39	72.84
84	Sh:Q-162 UR-17		35.22	−89.85	83.45	83.26	84.31	84.52
85	Sh:Q-163 Sh:UR-18		35.2	−89.83	66.13	65.79	63.39	63.06
86	Sh:Q-164 Sh:UR-19		35.18	−89.83	70.39	70.51	69.12	69.08
87	Sh:Q-166 UR-29		35.2	−89.81	67.48	67.05	64.70	64.14
88	Sh:Q-167 UR-30R		35.23	−89.82	71.39	71.41	69.39	70.01
89	Sh:Q-173		35.14	−89.85	68.2	68.44	65.63	65.39
90	Sh:Q-177		35.14	−89.85	68.95	69.22	66.58	66.68
91	Sh:Q-185 UR-28		35.19	−89.78	102.46	102.61	102.35	102.76
92	Sh:R-010	PR-MB-CAR	35.14	−89.66	83.15	83.01		86.58
93	Sh:R-032	7T2	35.14	−89.72	85.1	85.02	84.67	85.77
94	Sh:V-014	PR-KM-MCC	35.36	−89.78	95.44	92.35	91.35	91.68
95	Sh:V-018	PR-PT-BR	35.33	−89.76	89.12	89.12	88.59	88.70
96	Sh:W-014	PR-CA-PAL	35.3	−89.74	85.24	84.41	83.07	83.45
97		PR_BP_ISF	35.29	−90.04		70.43		
98		PR-JT-WU	35.37	−89.92	71.97	71.82	69.99	70.06
99		PR-DF-CVP	35.3	−89.85	79.78	78.89		
100		PR-BG-LJ	35.3	−89.97	73.94	73.3		
101		PR-GSP-BJT	35.28	−90.04		72.16		
102		NC-1	35.08	−89.93	71.22	70.62		
103		NC-2	35.08	−89.93	71.26	70.71		
104		NC-3	35.08	−89.93	71.24	70.78		
105		FRL-MW1	35.07	−89.69	85.87	85.7		
106		LITCO-1	35.04	−89.66	86.83	86.52		
107		FRL-TW	35.07	−89.69	82.73	82.37		
108		GG-MW1	35.1	−89.78	75.84	74.26		
109		S-4	35.08	−89.67	84.12	84.28		
110		S-5	35.08	−89.67	84.05	84.21		
111		SP-SHELL-RL	35.2	−89.98	73.62	73.62		
112		79561	35.26	−89.97		70.69		
113		79582	35.15	−89.97	69.34	70		
114		79755	35.05	−90.04		74.88		
115		79843	35.17	−89.96	71.19	69.29		
116		79852	35.16	−90.04	70.77	69.77		
117		79598	35.19	−89.98	66.06	65.29		
118		79552	35.04	−89.69	84.38			
119		79742	35.32	−89.91	89.71			
120		79799	35.07	−89.97	85.11			
121		79845	35.04	−89.65	87.37			
122		9790823	35.22	−89.93	90.3	89.52		
123		9790821	35.13	−90.02	80.44	79.53		
124		9790812	35.21	−90.03	73.58	71.85		
125		9792331	35.03	−89.83	94.97	94.84		
126		D79137	35.11	−89.89		69.96		
127		D79140	35.05	−89.91	60.17	60.15		
128		D79158	35.16	−89.94		75.48		
129		D79198	35.34	−89.88		76.8		
130		D79100	35.14	−90	78.49			
131		D79193	35.07	−89.84	78.69			
132		D79242	35.06	−89.89	60.91			
133		SRS790009	35.06	−90.05	73.63			
134		SRS790044	35.12	−90.08	64.65	64.36		
135		SRS790189	35.12	−90.07	66.1	65.92		
136		SRS790732	35.28	−89.95	70.6	69.69		
137		Former Custom Cleaners	35.11	−89.94	53.31	54.58		
138		SRS790825	35.06	−90	69.56	68.75		
139		BR-BCG-LJR	35.31	−89.98	66.83	62.67	62.51	62.64
140		BR-BCG-MVR	35.38	−89.81	89.81	89.67		
141		BR-BC-OBR	35.32	−89.66	74.95	74.5	74.42	74.72
142		BR-BGC-205	35.34	−89.81	82.6	82.36		82.92
143		BR-BGC-FTE	35.28	−90.01	66.83	61.29	61.12	61.10
144		BR-BGC-H51	35.33	−89.92	68.22	67.6	67.46	67.45
145		BR-BGC-KRR	35.37	−89.8	86.13	85.84	84.98	85.83
146		BR-BGC-NBS	35.32	−89.87	73.75	73.55	73.41	73.69
147		BR-BGC-SLG	35.33	−89.83	77.51	77.29		
148		BR-CC-AIR	35.22	−89.65	81.44	81.34	81.08	81.20
149		BR-CC-H70	35.27	−89.7	74.8	74.69	74.23	74.31
150		BR-CC-I40	35.25	−89.69	76.75	76.52	76.32	76.65
151		BR-CRC-APH	35.32	−89.81	79.29	79.12		
152		BR-CRC-MAR	35.34	−89.76	87.9	87.77	87.70	88.68
153		BR-CYP-MIT	35.06	−90.09	64.53	64.44	64.43	64.42
154		BR-FC-I40	35.19	−89.8	80.64	80.71	80.51	80.42
155		BR-FC-RLG	35.18	−89.84	72.01	72.03	71.83	71.93
156		BR-FC-SUM	35.17	−89.87	69.76	69.76		
157		BR-FC-WTN	35.19	−89.84	73.52	73.54	73.27	73.32
158		BR-GRC-RHR	35.2	−89.68	92.1	91.99		92.08
159		BR-GRC-WGR	35.13	−89.74	77.77	77.64	77.57	77.62
160		BR-HC-RLG	35.19	−89.89	69.58	69.47	69.37	69.38
161		BR-JC-CMR	35.36	−89.97	76.73	76.58	76.66	76.94
162		BR-JC-RBR	35.35	−89.97	73.67	73.52	73.04	
163		BR-LH-CVA	35.31	−89.67	74.08	73.47		
164		BR-LR-385	35.3	−89.69	73.22	72.53	72.33	72.32
165		BR-LR-70A	35.31	−89.64	75.73	75.38	75.07	75.15
166		BR-LR-APH	35.28	−89.85		67.56	67.41	67.40
167		BR-LR-BRW	35.28	−89.77	70.88	70.39		
168		BR-LR-H51	35.26	−89.99	66.83	60.76	61.29	61.38
169		BR-LR-RMR	35.26	−89.93	66.87	65.17		
170		BR-LR-SNG	35.28	−89.89	67.47	66.75	66.67	66.71
171		BR-LR-WTK	35.25	−90.03	66.77	59.01		
172		BR-NC-BY	35.04	−89.69	98.75	98.73		
173		BR-NC-EPB	35.07	−90.02	64.37	61.63	61.56	61.67
174		BR-NC-FAR	35.08	−89.95	67.86	67.84		
175		BR-NC-FH	35.03	−89.76	86.73	86.68		
176		BR-NC-GET	35.08	−89.93	71.52	71.48		
177		BR-NC-HC	35.04	−89.8	83.92	83.84	83.74	83.82
178		BR-NC-HL	35.03	−89.72	91.98	91.96	91.48	84.22
179		BR-NC-KBY	35.07	−89.84	78.66	78.57	78.55	78.61
180		BR-NC-NCB	35.07	−89.99	65.76	65.79		
181		BR-NC-NHL	35.08	−90.06	65.25	57.84	57.62	58.03
182		BR-NC-PER	35.08	−89.91	73.05	73.02		
183		BR-NC-RWY	35.07	−89.86	78.62	77.95	77.95	77.99
184		BR-NC-SYC	35.03	−89.68	102.39	102.36		
185		BR-NC-WIN	35.05	−89.82	82.09	82.05	81.72	81.70
186		BR-NFC-WR	35.39	−89.89	89.39	89.14	88.02	87.94
187		BR-OC-H70	35.25	−89.76	81.56	81.45		
188		BR-RC-UR	35.36	−89.91	75.78	75.49	75.47	75.57
189		BR-UKN-WGR	35.13	−89.76	84.68	84.69	84.67	84.97
190		BR-WBC-DFL	35.38	−89.72	88.58	88.49	88.33	88.89
191		BR-WBC-MOO	35.36	−89.7	85.26	85.15		
192		BR-WBC-OSB	35.36	−89.69	84.5	84.5	84.44	84.39
193		BR-WR-APH	35.2	−89.92	66.68	65.54		
194		BR-WR-CA	35.08	−89.65	84.8	84.41		83.70
195		BR-WR-CVP	35.17	−89.9	67.94	67.16	66.91	66.88
196		BR-WR-GT	35.12	−89.8	73.66	72.79	72.68	72.84
197		BR-WR-H51	35.19	−90.04	66.33	58.62	57.50	58.27
198		BR-WR-HIL	35.19	−89.94	66.37	64.08	63.68	63.75
199		BR-WR-HL	35.11	−89.73	77.02	76.12	75.60	75.52
200		BR-WR-HWD	35.19	−89.98	66.37	62.45	62.08	62.16
201		BR-WR-I40	35.19	−90.02	66.37	60.08	59.67	59.68
202		BR-WR-MCL	35.19	−89.99	68.58	61.38	60.99	60.96
203		BR-WR-SUM	35.16	−89.88	68.8	68.04	67.72	67.75
204		BR-WR-WG	35.13	−89.85	70.88	70.17	69.62	70.15
205		BR-WR-WRF	35.19	−89.96	66.38	63.38	62.94	63.05
206		BR-WR-WTK	35.19	−89.99	68.26	62.48	60.99	60.96
207		NC_UR13_SW	35.08	−89.88	73.51	73.12		
208		WR_GTOWN_SW	35.11	−89.78	74.89	74.21		
209		9790505					78.85	80.78
210		9790657					92.38	80.31
211		9790817					68.34	68.00
212		9790822					81.74	81.47
213		9791067					80.44	81.69
214		9791084					77.58	78.24
215		9791242					84.59	84.42
216		9791410					90.08	87.72
217		9791551					86.92	89.00
218		9791826					92.99	92.78
219		9791911					74.83	73.81
220	Sh:J-193	4T2					72.72	73.17
221	Sh:K-03-MW17						65.65	65.49
222	Sh:P MW-15B						69.45	69.13
223	Sh:P MW-19D						69.71	67.39
224	Sh:P-121						64.00	62.73
225	Sh:P-122						66.09	65.26
226	Sh:P-135						63.90	63.09
227	Sh:P-136						66.56	65.19
228	Sh:P-138						64.35	63.19
229	Sh:P-148						89.84	90.04
230	Sh:P-150						66.11	65.05
231	Sh:P-151						63.79	63.11
232	Sh:P-152						66.63	65.51
233	Sh:P-157						66.85	66.33
234	Sh:P-158						66.66	66.32
235	Sh:P-160						66.55	66.26
236	Sh:P-161						66.51	66.33
237	Sh:P-174						65.61	64.11
238	Sh:P-175						65.54	64.09
239	Sh:P-188						64.89	64.36
240	Sh:Q-116						69.62	70.11
241	Sh:T-009						97.92	98.38
242	Sh:T-019						69.92	70.18
243	Sh:U-035						85.85	87.18
244	Sh:U-205						83.14	80.72
245	Sh:UR-09						78.44	77.43
246	Sh:V-022						70.86	71.39
247		79208					77.69	
248		79210					81.32	
249		79219					71.12	
250		79235					72.62	
251		79514					88.07	
252		79582					70.17	
253		79742					74.44	
254		79758					67.22	
255		79786					73.09	
256		79802					75.86	
257		9790081					68.21	
258		9790132					64.47	
259		9790178					72.69	
260		9790314					70.12	
261		9790455					69.24	
262		9790486					77.55	
263		9790497					76.85	
264		9790504					65.74	
265		9790618					79.53	
266		9790641					68.93	
267		9790642					81.46	
268		9790781					79.72	
269		9790813					66.80	
270		9790815					76.73	
271		9790827					81.20	
272		9791070					77.62	
273		9791083					76.22	
274		9791093					68.31	
275		9791239					79.10	
276		9791367					72.75	
277		9791374					76.22	
278		9791379					75.03	
279		9791392					79.96	
280		9791394					70.68	
281		9791419					73.70	
282		9791422					82.81	
283		9791439					70.23	
284		9791539					77.11	
285		9791613					71.45	
286		9791618					99.66	
287		9791654					65.83	
288		9791951					79.85	
289		9792055					83.39	
290		9792088					74.08	
291		9792141					66.49	
292		9792547					85.10	
293		9792674					90.69	
294		9792713					78.81	
295		BFI-1					75.94	
296		BFI-10					63.99	
297		BFI-11					63.00	
298		BFI-12					74.11	
299		BFI-13					79.30	
300		BFI-14					77.08	
301		BFI-15					75.27	
302		BFI-18					73.74	
303		BFI-19					62.62	
304		BFI-2					78.48	
305		BFI-20					62.52	
306		BFI-8					63.10	
307		BFI-9					63.70	
308		DR2-2					69.15	
309		IW101-03C					62.56	
310		IW92-07					64.57	
311		MW-197A					61.50	
312		MW-212					61.41	
313		MW-85					63.56	
314	Sh:M-060						85.29	
315		SRS-1268					67.78	
316		SRS790295					63.67	
317		8000 Mill-Arlington						80.90
318		2705 Little John						70.13
319		8310 Hwy 14						91.40
320		9790237						75.14
321		9790481						64.08
322		9790547						69.75
323		9790625						82.26
324		9790818						58.25
325		9791085						69.95
326		9791087						75.39
327		9791219						76.04
328		9791220						75.76
329		9791241						83.58
330		9791244						89.96
331		9791246						70.84
332		9791259						77.30
333		9791271						85.99
334		9791431						74.48
335		9791530						70.13
336		9791566						76.83
337		9791584						81.13
338		9791543						78.69
339		9791102						60.59
340		9791630						68.59
341		9791861						83.03
342		9792153						58.13
343		9792223						92.45
344		9792369						82.20
345		9792583						101.50
346		br_33						67.75
347		br_34						68.54
348		br_40						82.77
349		br_43						94.48
350		br_64						92.88
351		br_74						98.85
352		ddmt MW-182						65.18
353		ddmt MW-185						55.25
354		Sh: DH-3						80.09
355		Sh: P/MW-3						78.92
356	SH:J-210							74.48
357	Sh:K-020							71.91
358	SH:K-129	8T3						98.63
359	SH:K-157							95.69
360	SH:L-052							75.69
361	Sh:L-114							67.85
362	Sh:M- Standard							82.34
363	Sh:M-061							82.12
364	Sh:M-064							81.33
365	SH:O-214							65.06
366	SH:O-252-b							56.73
367	Sh:P MW-17							68.28
368	SH:Q-079							74.51
369	SH:Q-086							72.70
370	SH:Q-093							82.75
371	Sh:Q-095							69.51
372	Sh:Q-104							71.79
373	Sh:Q-119							65.16
374	SH:Q-131							94.09
375	Sh:Q-136							68.55
376	SH:T-021							95.11
377	SH:T-024							75.87
378	Sh:U-083							72.00
379	Sh:U-087							97.40
380	Sh:U-138							76.82
381	Sh:U-197							71.79
382	Sh:U-206							65.35
383	SH:UR-31							63.75
384	Sh:V-013							80.43
385	SH:V-019							81.46
386	Sh:V-108							78.91
387	Sh:V-262							74.66
388	Sh:V-263							76.59
